# Ranolazine as an Alternative Therapy to Flecainide for SCN5A V411M Long QT Syndrome Type 3 Patients

**DOI:** 10.3389/fphar.2020.580481

**Published:** 2020-12-17

**Authors:** Jordi Cano, Esther Zorio, Andrea Mazzanti, Miguel Ángel Arnau, Beatriz Trenor, Silvia G. Priori, Javier Saiz, Lucia Romero

**Affiliations:** ^1^ Centro de Investigación e Innovación en Bioingeniería (CI2B), Universitat Politècnica de València, Valencia, España; ^2^ Unidad de Cardiopatías Familiares y Muerte Súbita, Servicio de Cardiología, Hospital Universitario y Politécnico La Fe, Valencia, España; ^3^ Center for Biomedical Network Research on Cardiovascular Diseases (CIBERCV), Madrid, Spain; ^4^ Molecular Cardiology, IRCCS, Istituti Clinici Scientifici Maugeri, Pavia, Italy; ^5^ Department of Molecular Medicine, University of Pavia, Pavia, Italy

**Keywords:** Long QT Syndrome, flecainide, ranolazine, in-silico model, sodium current channelopathy, V411M

## Abstract

The prolongation of the QT interval represents the main feature of the long QT syndrome (LQTS), a life-threatening genetic disease. The heterozygous SCN5A V411M mutation of the human sodium channel leads to a LQTS type 3 with severe proarrhythmic effects due to an increase in the late component of the sodium current (INaL). The two sodium blockers flecainide and ranolazine are equally recommended by the current 2015 ESC guidelines to treat patients with LQTS type 3 and persistently prolonged QT intervals. However, awareness of pro-arrhythmic effects of flecainide in LQTS type 3 patients arose upon the study of the SCN5A E1784K mutation. Regarding SCN5A V411M individuals, flecainide showed good results albeit in a reduced number of patients and no evidence supporting the use of ranolazine has ever been released. Therefore, we ought to compare the effect of ranolazine and flecainide in a SCN5A V411M model using an in-silico modeling and simulation approach. We collected clinical data of four patients. Then, we fitted four Markovian models of the human sodium current (INa) to experimental and clinical data. Two of them correspond to the wild type and the heterozygous SCN5A V411M scenarios, and the other two mimic the effects of flecainide and ranolazine on INa. Next, we inserted them into three isolated cell action potential (AP) models for endocardial, midmyocardial and epicardial cells and in a one-dimensional tissue model. The SCN5A V411M mutation produced a 15.9% APD90 prolongation in the isolated endocardial cell model, which corresponded to a 14.3% of the QT interval prolongation in a one-dimensional strand model, in keeping with clinical observations. Although with different underlying mechanisms, flecainide and ranolazine partially countered this prolongation at the isolated endocardial model by reducing the APD90 by 8.7 and 4.3%, and the QT interval by 7.2 and 3.2%, respectively. While flecainide specifically targeted the mutation-induced increase in peak INaL, ranolazine reduced it during the entire AP. Our simulations also suggest that ranolazine could prevent early afterdepolarizations triggered by the SCN5A V411M mutation during bradycardia, as flecainide. We conclude that ranolazine could be used to treat SCN5A V411M patients, specifically when flecainide is contraindicated.

## Introduction

Mutations in genes encoding ion channels are regarded as the molecular basis of the congenital Long QT Syndrome (LQTS). Abnormal proteins expressed at the heart result in disrupted electrical activity that pose the patients at an increased risk of lethal arrhythmias such as Torsade-de-pointes. Seventeen types of congenital LQTS with autosomal dominant inheritance pattern and 2 types of autosomal recessive LQTS have been described so far. Five of them affect the activity of the sodium current (INa) (Bohnen et al., 2017) and also account for 5–10% of the cases (Ackerman et al., 2011). Mutations in the SCN5A gene (LQTS type 3), which encodes the main protein of INa, and mutations in the genes encoding its regulatory proteins (LQTS 4, LQTS 9, LQTS 10 and LQTS 12) can in fact cause LQTS as long as the mutations result in a gain-of-function of the late sodium current. Current clinical guidelines and independent research papers recommend beta-blockers and may consider the addition of several drugs such as ranolazine, mexiletine, flecainide or lidocaine when treating LQTS type 3 patients with QTc over 500 ms (Priori et al., 2015; Bengel et al., 2017; Bohnen et al., 2017). However, some concern was raised when the SCN5A overlap syndrome was identified in patients exhibiting not only a LQTS type 3 phenotype but also Brugada syndrome and conduction defect features. In them, high temperatures and/or the administration of flecainide could unmask the other phenotypes and trigger life-threatening arrhythmias (Makita et al., 2008; Abdelsayed et al., 2015). Basic research on these drugs has revealed that drug-channel interaction is complex and not just limited to a specific blockade of part of INa, the fast (INaf) or the late (INaL) components.

Patients with the V411M mutation in the SCN5A gene present congenital LQTS type 3 with symptoms very early in life and, if left untreated, can lead to severe arrhythmia (Carrasco et al., 2012; Blich et al., 2019). The clinical beneficial effects of flecainide on ventricular arrhythmia management and on QTc measurements in patients with LQTS type 3, specifically those harboring the V411M mutation, is consistent with its blocking effect on INa (Carrasco et al., 2012; Blich et al., 2019). It has been hypothesized that these positive outcomes rely on a high affinity binding and subsequent blocking of the open state of the sodium channel. Flecainide is a class IC antiarrhythmic drug because of its ability to block sodium channels and it is effective both for cardioversion as well as sinus rhythm maintenance in atrial fibrillation (Aliot et al., 2011). It has been used in the clinical practice for a long time—in Europe since 1982—while showing a good safety profile if correctly monitored (Aliot et al., 2011). By contrast, lidocaine showed no effect on a patient with the V411M heterozygous mutation (Horne et al., 2011). Ranolazine is a relatively new class IB antiarrhythmic drug with selective I_NaL_ block that has shown effectiveness in the treatment of angina pectoris and in patients with LQTS caused by the D1790G mutation (Chorin et al., 2016) but, to the best of our knowledge, it has not been tested in patients with V411M mutation.

Mathematical models are innovative tools that can provide useful insights into the mechanisms that drive the electrical activity of the heart. Markov models of the ion channels are specially indicated to study ion channels gating and their interaction with drugs. They describe the different states that channels can occupy, which correspond to different channel conformations. Transitions between these states are described through detailed descriptions of time and voltage-dependent transition velocities (Rudy and Silva, 2006). They have already been used to model and test the effects of drugs in multiple scenarios, including mutated cardiomyocytes. For example, drugs affecting the rapid component of the delayed rectifier potassium current (I_Kr_) have been modeled to enable a better understanding of their interactions (Romero et al., 2015; Li et al., 2017; Yang et al., 2020), and have been successfully validated for proarrhythmia assessment (Chang et al., 2017) at the core of the in-silico section of the Comprehensive *In-vitro* Proarrhythmia Assay initiative (CiPA). Similarly, Moreno et al. (Moreno et al., 2013) modeled both I_Na_ and SCN5A ΔKPQ, a LQTS type 3-inducing mutation that prolongs the action potential duration (APD) of cardiac cells by increasing the sustained component of I_Na_, creating the perfect conditions for early afterdepolarizations (EADs) occurrence. Moreover, these authors proposed mathematical formulations for flecainide, ranolazine and lidocaine and tested their effects in catecholaminergic polymorphic ventricular tachycardia serving as a proof-of-concept of a multi-drug treatment (Yang et al., 2016a).

It is well known that I_NaL_ is a key component in the regulation of the APD (Saint, 2009) especially in conditions of specific heart diseases, such as heart failure and angina (Moreno and Clancy, 2012; Trenor et al., 2012; Makielski, 2016; Chadda et al., 2017). However, this component of the sodium current is not easy to characterize. Recently, new experimental methods to characterize I_NaL_ have been proposed. Indeed, to better capture the dynamics of I_NaL_ during the AP and its role in arrhythmogenesis, works by Horvath et al. (2013), Hegyi et al. (2018) used self-AP clamp techniques to characterize the current’s time course instead of using the traditional single rectangular pulse. In addition, Li et al. (2017) found that I_NaL_ was one of the three main currents whose block should be taken into account to improve drug proarrhythmicity prediction when simulating the electrophysiological effects of drug-channel interactions.

Herein, we attempt to shed light into the mechanisms behind the heterozygous SCN5A V411M mutation phenotype and to study the potential of ranolazine to reduce its abnormal behavior as an alternative to flecainide by using computer simulations. Moreover, we gained insights into the mechanisms of these two drugs to mitigate the mutation phenotype. To do so, first, we improved the well-known (Moreno et al., 2013) I_Na_ model by adjusting it to new I_NaL_ data and generated a new model for the V411M mutation. Then, we refitted the models for both drugs, flecainide and ranolazine, while improving the former with new tests, to evaluate their potential therapeutic effects at several pacing rates both on isolated cell and tissue model conditions. Finally, we explored the compounds’ potential to prevent arrhythmia triggers during bradycardia. This study represents one step forward to the implementation of computational tools fulfill the promise of personalized and precision medicine.

## Materials and Methods

### Markovian Sodium Channel Model

We optimized the wild-type sodium channel model from (Moreno et al., 2013), as well as the updated version of flecainide including bursting states (Yang et al., 2016b) and ranolazine (Moreno et al., 2013) to reproduce additional experimental data (Penniman et al., 2010; Horvath et al., 2013). These are part of a framework that focuses on facilitating the process of combining experimental data into functional ion channel models (Moreno et al., 2016). Their versatility, combined with their highly detailed formulation based on works that span more than 20 years of research, represents a suitable substrate for our objectives. We generated a V411M mutated model that can be set in homo or heterozygous configuration from the updated wild-type version. Simulation of the heterozygous V411M mutation was modeled by combining 50% wild type and 50% mutant currents, as in Moreno et al. (2013). The homozygous model was used in *in-silico* patch-clamp experiments while the heterozygous model was used to simulate action potentials (APs). All I_Na_ models were constrained by microscopic reversibility (Colquhoun et al., 2004).

### Isolated Cellular Action Potential Models

We used the Grandi et al. (2010) modified with Soltis and Saucerman (2010) by Moreno et al. (2013) epicardial model as a starting point. Then, we modified the current conductances (O'Hara et al., 2011) to create endocardial and midmyocardial versions, which were fitted to their respective APD at 90% repolarization (APD_90_) restitution curves (see Supplementary Figure S1). We gathered the specific changes in conductances between cell types in Supplementary Table S1. In order to reduce computational demand of our model fits, we first used the APD of the endocardial cellular model as a surrogate of the QT interval, as in a previous work of our group (Romero et al., 2018) we found that risk prediction based on 10% of the human endocardial APD_90_ and QT prolongations gave very similar results.

### Transmural Wedge Model

The ventricular transmural wedge model consisted of 165 cells connected in a row, split in three zones of 60, 45 and 60 endocardial, midmyocardial and epicardial cells, respectively. Stimulation was applied to the first endocardial cell and propagation through the strand was simulated using the following nonlinear reaction diffusion equation:

$$C_{m}\cdot \frac{\partial v_{m}\left(x,t\right)}{\partial t}+\Sigma I_{ion}+\frac{a}{2}\cdot \frac{\partial }{\partial x}\cdot \left(\frac{1}{R_{i\left(x\right)}}\cdot \frac{\partial V_{m\left(x,t\right)}}{\partial x}\right)=0$$

where $C_{m}$ is the membrane capacitance, “a” stands for the radius of the fiber, “$\Sigma I_{ion}$” represents the sum of all the ionic currents flowing through the cellular membrane, and “$R_{i}$” is the intracellular resistivity.

The inclusion of a virtual electrode 2 cm away from the epicardial end enabled the calculation of the pseudo-electrocardiogram (ECG) of the strand, although the first and last 15 cells were not taken into account for this purpose to prevent boundary effects.

### Simulation Protocols

Unless specified, we paced the models at a rate of 1 Hz, a frequency that is widely used in human cardiomyocyte simulation (Elkins et al., 2013; Crumb et al., 2016; Lancaster and Sobie, 2016) in the absence of β-adrenergic stimulation. These conditions would be equivalent to the exposure to a β-blocker drug. The single cellular model was stimulated with square pulse of −9.5 pA/pF and 5 m duration. The model was simulated with a custom C++ script. State occupancy probabilities in the I_Na_ Markov model were calculated by an implicit Trapezoidal numerical method. The numerical method used to update the voltage was forward Euler.

Patch clamp simulations of the I_Na_ channel were simulated using a custom Matlab (The Mathworks, inc.) script including an ordinary differential equation solver with variable time step.

The ventricular transmural wedge model was stimulated with a −400 pA/pF square pulse of 0.5 m applied to the first endocardial cell. The numerical method used to simulate the model was Forward Euler with a fixed time step of 0.005 m and it was run using a customized C++ code.

Both ranolazine and flecainide showed evidence of blocking the rapid component of the delayed rectifier potassium current (I_Kr_). We simulated it by using the Hill formalism, consisting of the following equation:

$$f_{o}=\frac{1}{1+{\left(\frac{C}{IC_{50}}\right)}^{H}}$$

Where $f_{o}$ is remaining conductance of the channel at a concentration C of a drug exhibiting a half-maximal block concentration for the channel of $IC_{50}$, and modulated by H, the Hill coefficient, which was set to 1 as in our previous work (Romero et al., 2018).

In order to reduce computational cost, simulation of the APD_90_ restitution curve in wild type, the prolongation in V411M mutation and the prolongation in flecainide were performed using the following procedure. We first paced the original Moreno et al. (2013) I_Na_ model (Moreno et al., 2013) for 300 pulses for each case and the state variables were saved. Then, they were used as initial conditions in the optimization procedure and a train of 40 pulses was applied to the isolated endocardial cell models for each iteration.

Details of the methods including patch clamp, simulation and optimization protocols are available in the Supplementary Material of this work.

### Clinical Characterization

Two dedicated clinics with expertise in channelopathies enrolled LQTS patients heterozygous for the SCN5A V411M mutation. Exclusion criteria was “not willing to take part in the study.” The study protocol was approved by the local ethics committee. The QT interval was manually measured with the tangent method (Postema et al., 2008) in at least three consecutive complexes from lead II, unless the quality of the ECG or the presence of pronounced sinus arrhythmia made it reasonable to reduce the number of complexes to two or choose nonconsecutive beats to gain accuracy, never looking for the longest or shortest QT intervals. The freely accessible online probability calculator for LQTS^1^ was used to obtain the QT interval value with Bazzet correction (QTc) and a mean value was calculated from resting 12-lead ECGs on beta-blocker treatment and, if available, also naïve. Notably, the website provides the Gaussian percentiles of the QTc interval in controls of similar sex and age, allowing us to identify the specific QTc value below which 90% of the observations in controls (90th percentile) can be found. Being very conservative, we took that value as the reference in general population to calculate the magnitude of the prolongation caused by the SCN5A V411M mutation in each patient and a mean value for that effect (to that end, only naïve ECG were taken into consideration).

## Results

### QTc Measurements in Patients

**FIGURE 1:**
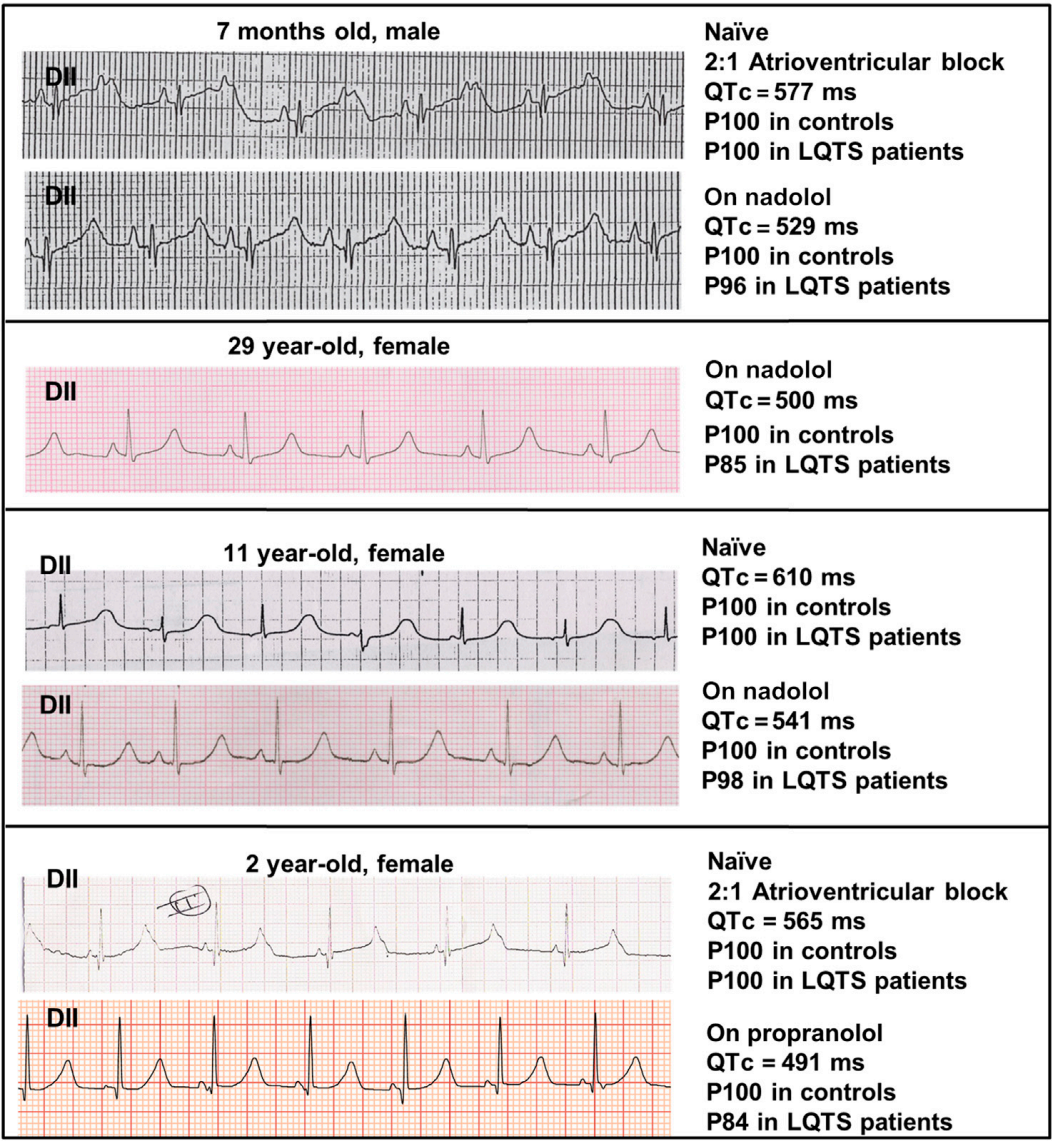
ECG recordings from our patients. Lead II recordings from the ECGs of four patients with the heterozygous SCN5A V411M mutation, along with information about their measured QTc values in naïve (without treatment) and under β-blocker treatment (nadolol or propranolol). A “P” precedes the percentile to which QTc values belong.

Figure 1 shows a lead II from the ECGs obtained in the four patients depicting the QTc value and the estimated probability of finding such a value among controls and LQTS patients. As observed, naïve QTc intervals were significantly prolonged and well above the well-known cutoff of 500 ms and the two youngest patients additionally exhibited atrioventricular block 2:1. The use of beta-blockers could only mildly ameliorate QTc values. In order to estimate the QTc prolongation attributable to the SCN5A V411M mutation, we considered the QTc of patients with beta-blockers as our reference since our models were ran without accounting for beta stimulation. Two QTc values were obtained from each of four patients, as described in Table 1. The QTc increments ranged from 8.9 to 28.1% and the average was 19.9%.

**TABLE 1:** Patients information.

Patient	Gender	Age (years)	β-blocking Drug	QTc (ms)	QTc leaving 90% of control QTc below (ms)	QTc increment (%)
1	Male	1	Nadolol	542	434	24.9
		(7 months)		516	434	18.9
2	Female	29	Nadolol	503	437	15.1
				498	437	14.0
3	Female	11	Nadolol	555	434	27.9
				528	434	21.7
4	Female	2	Propranolol	471	434	8.5
				556	434	28.1
					Average	19.9

This table contains anonymized relevant data of our four patients (from 1 to 4), namely, from left to right, their assigned code, gender, age (in years), the name of the administered β-blocking drug, registered QTc interval values (in ms), reference QTc values (in ms) and calculated QTc increment (in %). The last row shows the average QTc increment.

### Models

To evaluate the usefulness of either flecainide or ranolazine as treatments for the LQTS type 3 syndrome caused by the SCN5A V411M mutation, we selected the Grandi-Bers with Soltis-Saucerman human epicardial cell model (GBers-SS) modified by Moreno et al. (2013) as a starting point because it includes their Markovian sodium channel formulation.

We modified several GBers-SS ionic current conductances following the same procedure as in O’Hara et al. (2011) to generate endocardial and mid-myocardial versions from the epicardial one. Then, we fitted the wild type sodium current model using the endocardial version of the human ventricular action potential. Next, we tuned it to mimic the effects of the mutation, and finally, we adapted the flecainide model to additional experimental data and updated the ranolazine model for the new wild type model.

### Wild Type Sodium Current Optimization Results and Analysis

**FIGURE 2:**
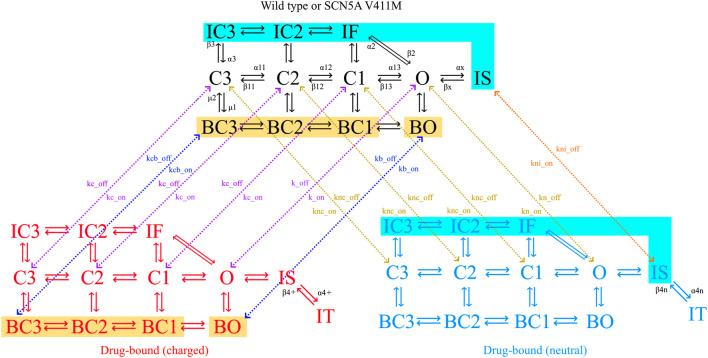
Diagram of the sodium channel Markovian model. The channel can be in closed (C), inactivated (I) or open (O) states as indicated by their name. The model has fast (IF, IC2, IC3) and slow (IS) inactivation dynamics, as well as a bursting mode of operation (B). Flecainide (but not ranolazine) trapping dynamics are represented by the trapped state (IT). Transitions between states are indicated with solid arrows, together with their respective transition rates. Drug-free states are depicted in black (wild type or mutated), charged drug-bound channels in red and neutral drug-bound states in blue. Binding (on) and unbinding (off) rates (k) determine the transitions between models, shown with dotted arrows. Color-coded zones indicate channel states that are connected to each other, but their arrows have been omitted for clarity. Yellow zones highlight the bursting states where the charged drug the binds and unbinds, the transition rates being kbc_on and kc_on for the binding and kbc_off and kc_off for the unbinding. Cyan zones highlight the inactive states where the neutral drug the binds and unbinds, the transition rates being ki_on for the binding and ki_off for the unbinding.

The sodium channel formulation proposed by Moreno et al. (2013) includes 12 states describing its precise conformations which include three closed, four inactive, four bursting and one open state. Transition rates between states are voltage dependent, adding to the complexity of the model and contributing to its ability to fit given dynamics. The bursting state mode (four states indicated with a B) represents the portion of the channels that are unable to correctly inactivate, contributing to the current’s late component. This feature, which was added later during the model development, shows that new features can be added in the future, if necessary, to account for new discoveries. We depicted the precise states and state transition velocities of the sodium current model in Figure 2.

Making use of the inter-experimental compatibility of Moreno and coworkers framework, we created a batch of tests designed to re-fit their Markovian I_Na_ model (Moreno et al., 2013). These tests considered its current-voltage relationship as (Moreno et al., 2011), its time course, and the isolated endocardial APD_90_ restitution curve. In order to fit the time course of the sodium current, we used the experimental data provided by Horvath et al. (2013), who employed a self-AP clamp protocol for that purpose.

We started our fitting from Moreno et al. (2013) optimal parameters, which reproduce very well the current-voltage relationships. In order to overcome the local minimum generated by this fact, we divided the wild type optimization into two phases. Phase one simulated the endocardial APD_90_ restitution, current time course and maximum upstroke velocity (max dV/dt). Phase two took into account the original patch clamp protocols used by Moreno et al., 2011, which assess steady-state availability, activation, recovery from inactivation, recovery from use-dependent block and mean opening time dynamics. The total charge carried by I_Naf_ (qNaf) was also controlled to prevent the model from not depolarizing. Parameters and equations of the final wild type model are shown in Table 2.

**TABLE 2:** Transition rates of the post-optimization wild type model.

States	Transition rates
IC3 →IC2, C3→C2, BC3→BC2	α11 = Tf × 8.5539/(6.59e-2×exp(−v/(17.0))+2.76e-1×exp(−v/(150)))
IC2→IF, C2→C1, BC2→BC1	α12 = Tf × 8.5539/(6.59e-2×exp(−v/(15.0))+2.76e-1×exp(−v/(150)))
C1→O, BC1→BO	α13 = Tf × 8.5539/(6.59e-2×exp(−v/(12.0))+2.76e-1×exp(−v/(150)))
IC2→IC3, C2→C3, BC2→BC3	β11 = Tf × 6.98e-2×exp(−v/20.3)
IF→IC2, C1→C2, BC1→BC2	β12 = Tf × 3.04×exp(−(v-5)/20.3)
O→C1, BO→BC1	β13 = Tf × 1.18×exp(−(v-10)/20.3)
IC3→C3, IC2→C2, IF→C1	α3 = Tf × 8.06e-6×exp(−v/(8.43))
C3→IC3, C2→IC2, C1→IF	β3 = Tf × 6.45×exp((v)/(1.48e1))
O→IF	α2 = Tf × 3.54×exp(v/(3.81e1))
IF→O	β2 = (α13×α2×α3)/(β13×β3)
O→IS	βx = 2.30e-2×α3
IS→O	αx = 3.02e-2×α2
C3→BC3, C2→BC2, C1→BC1, O→BO	µ1 = 1.70e-07
BC3→C3, BC2→C2, BC1→C1, BO→O	µ2 = 5.66e-04

The first column indicates state transitions, while the second column specifies their corresponding transition rate names and equations. “Tf” is a temperature scaling factor calculated from a Q10 of 3 as described in the Supplementary Material of this work. Nomenclature for transition rates and affinities: charged drug (+), neutral drug (n), closed states (c), inactivated states (i), bursting states (b), drug binding to the channel (on), drug unbinding from the channel (off).

**FIGURE 3:**
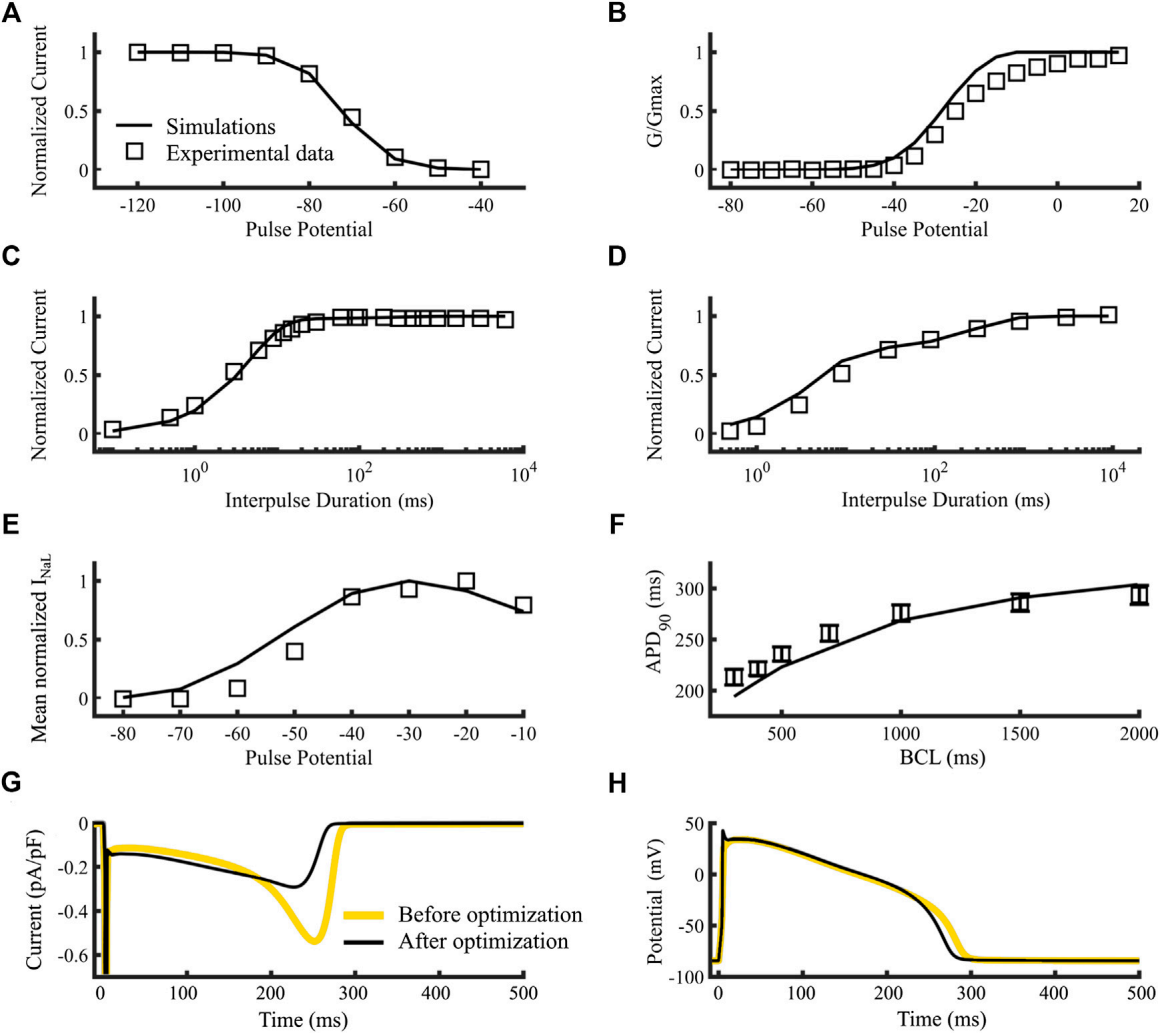
Optimization of the control model of the sodium current dynamics. Squares are references and lines are simulations **(A–F)**. **(A)** Steady-state availability. **(B)** Steady-state activation. **(C)** Recovery from inactivation. **(D)** Recovery from use-dependent block. **(E)** I_NaL_ current-voltage relationship. **(F)** APD_90_ Restitution curve of an isolated endocardial cell. **(G)** time course of the sodium current’s slow component of an isolated endocardial cell. **(H)** time course of the membrane potential of and isolated endocardial cell. Simulations using the original Moreno et al., 2013 model (yellow lines) and our new version (black lines) **(G,H)**. Data are from Rivolta et al. (2001) and Liu et al. (2002) **(A–D)**, O’Hara et al. (2011) **(E)** and Maltsev et al. (1998) **(F)**.

Figure 3 compares the simulation results of the wild type sodium current model dynamics (black lines) to experimental data (A-F, open squares). Figures 3A–D show the steady state availability, steady state activation, recovery from inactivation and recovery from use-dependent block, respectively. Figures 3E–H show the simulated I_NaL_ current-voltage relationship, APD_90_ restitution curve of endocardial cell, the current’s time course, and the membrane potential time course, respectively. Although the new model shows some small differences with the experimental data in the steady-state activation (Figures 3B) such as a higher slope and a 10-mV left shift of the activation curve, they are nonetheless within the range of commonly observed activation dynamics from several sources (see Supplementary Figure S3). Figures 3F shows that the simulated APD_90_ values at 300, 400, 500, 1,000, 1,500 and 2000 ms Basic Cycle Lengths (BCLs), were respectively, 194.2, 209, 223.1, 268.6, 290.9 and 303.9 ms (open squares with SD bars are experimental references, lines are simulations). The resulting inward dome current was 0.291 pA/pF, consistent with Li et al. (2017), the valley-to-dome current proportion was 48.0% and the half time proportion was 59.3%, which are in concordance with Horvath et al. (2013). A comparison between the sodium current time courses obtained with the original I_Na_ model (gray line) and our new version (black line) reveals a slight increase in plateau-phase and a reduction in repolarization phase peak. The corresponding action potential time courses were included in Figures 3H. The new model shortened the APD as a consequence of the reduction in late current during repolarization. The new max dV/dt (not shown) was 289.9 V/s compared to 250 V/s (O'Hara et al., 2011). Finally, the new late-to-fast current proportion was 0.08%, which is close to 0.1%, calculated following the methods used by Clancy et al. (2002). Overall, the post-optimization results show good agreement between simulation and experiments.

### SCN5A V411M Mutation Optimization Results

To fit the LQTS type 3 SCN5A V411M mutation model we constructed a batch of tests including specific patch clamp protocols to characterize the channel’s activation, inactivation, inactivation time constants, current-voltage relationship, and QT prolongation. Parameters and equations of the final V411M model are shown in Table 3. We performed a sensitivity analysis of the V411M mutation’s parameters to get some insights about how the differences between wild type and the mutation parameters are related to the increment of I_NaL_ (see Supplementary Figure S5A, Supplementary Table S2). The results of the analysis suggest that the modifications that most increased I_NaL_ were the decrease of β13 (deactivation, modulated by p5), followed by an increase of α3 (recovery from inactivation, modulated by p6 and p7).

**TABLE 3:** Transition rates of the post-optimization SCN5A V411M mutation model.

States	Transition rates
IC3 →IC2, C3→C2, BC3→BC2	α11 = Tf × 8.5539/(4.71e-2×exp(−v/(17.0))+2.52e-1×exp(−v/(150)))
IC2→IF, C2→C1, BC2→BC1	α12 = Tf × 8.5539/(4.71e-2×exp(−v/(15.0))+2.52e-1×exp(−v/(150)))
C1→O, BC1→BO	α13 = Tf × 8.5539/(4.71e-2×exp(−v/(12.0))+2.52e-1×exp(−v/(150)))
IC2→IC3, C2→C3, BC2→BC3	β11 = Tf × 5.65e-2×exp(−v/20.3)
IF→IC2, C1→C2, BC1→BC2	β12 = Tf × 2.99×exp(−(v-5)/20.3)
O→C1, BO→BC1	β13 = Tf × 2.59e-1×exp(−(v-10)/20.3)
IC3→C3, IC2→C2, IF→C1	α3 = Tf × 1.42e-5×exp(−v/(9.48))
C3→IC3, C2→IC2, C1→IF	β3 = Tf × 6.96×exp((v)/(1.67e1))
O→IF	α2 = Tf × 7.01×exp(v/(3.16e1))
IF→O	β2 = (α13×α2×α3)/(β13×β3)
O→IS	βx = 2.08e-2×α3
IS→O	αx = 5.51e-2×α2
C3→BC3, C2→BC2, C1→BC1, O→BO	µ1 = 1.70e-07
BC3→C3, BC2→C2, BC1→C1, BO→O	µ2 = 5.66e-04

The first column indicates state transitions, while the second column specifies their corresponding transition rate names and equations. “Tf” is a temperature scaling factor calculated from a Q10 of 3 as described in the Supplementary Material of this work.

**FIGURE 4:**
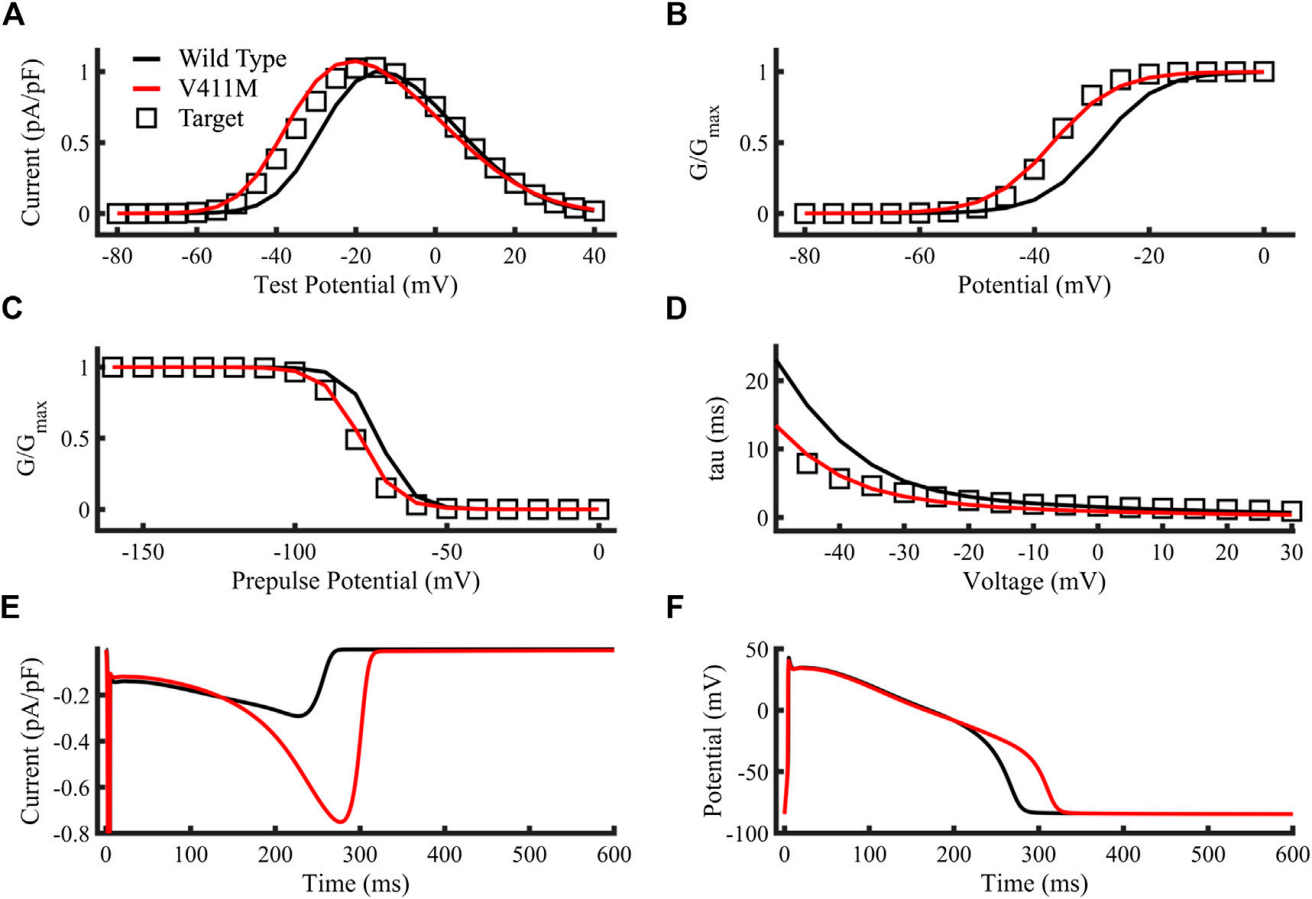
Optimization of the SCN5A V411M mutation model. Targets were represented as open squares, simulations using our SCN5A V411M mutation model as red lines and simulations using our wild type model as black lines. **(A)** Current-voltage relationship. **(B)** Steady state activation. **(C)** Steady state inactivation. **(D)** Inactivation time constants. **(E,F)** Simulated isolated endocardial time course of the sodium current’s slow component and the action potential for wild type (black line) and for heterozygous SCN5A V411M mutated (red line) cells. APD_90_ prolongation caused by the heterozygous mutation was a result of an increase in I_NaL_ as observed in its time course in **(E)** and was calculated by comparing wild type and mutation action potential time courses in **(F)**.

Figures 4A–D compare the targets (open squares) obtained from experimental results (Horne et al., 2011), the simulated V411M mutation (red lines) and the simulated WT current-voltage relationship, steady state activation, steady state inactivation and inactivation time constants (black lines), respectively. Protocols are described in Horne et al. (2011). The resulting V411M mutation model fitted correctly the target values, showing increased currents for test potentials from −60 mV to almost 0 mV, doubling the wild-type current for −40 mV to −30 mV test potentials, which are within the repolarization phase of the AP. The V411M mutated model produced a left shift of the curve half-maximal activation (V_1/2_) of 8.1 mV (Figures 4B), a successful fit to the reference 8.1 mV (Horne et al., 2011). The V411M model produced a left shift on the V_1/2_ of 6.1 mV and a slope increase of a 13.3%, consistent with the 7.9 mV and 13% target values (Horne et al., 2011), respectively (Figures 4C). Figures 4D depicts the reduction in inactivation time constants at test potentials from −50 mV to 30 mV. Inactivation time constants successfully diminished for the first portion of the curve (voltages from −50 mV to −20 mV) showing good agreement with the reference (Horne et al., 2011). Figures 4E,F show the simulated sodium current and the membrane voltage time courses for both wild-type (black line) and heterozygous mutation (red line) isolated endocardial cell simulations, respectively. I_NaL_ increased from a maximum value of 0.29 pA/pF in wild type to 0.75 pA/pF in the repolarization phase of the action potential producing a prolongation of the APD_90_ to 311.5 ms, or a 15.9% compared to wild type (268.5 ms), despite a slight decrease in the early plateau current. Therefore, our results show that the simulated ionic and cellular dynamics were close to the experimental and clinical observations.

### Drug Models

#### Flecainide

It is well known that flecainide produces an 8% prolongation of the QT segment in healthy patients.^2^ However, between a 60 to a 90% of that prolongation can be caused by the beforementioned QRS widening. Flecainide also blocks hERG channels (Paul et al., 2002) and increases the conductance of the potassium rectifier current I_K1_ (Caballero et al., 2010). To simulate the effects of therapeutic concentrations of flecainide (1.5 µM), we applied a 27.7% reduction of hERG conductance (Paul et al., 2002), which results from the Hill formalism with an IC_50_ of 3.91 µM and a Hill coefficient of 1, and increased I_K1_ conductance by a 51% according to published formula by Caballero et al. (2010).

We split the optimization of flecainide in three sequential phases with increasing number of tests. First, since the drug has a high pKa of 9.3 (Moreno et al., 2011), meaning it is 99% charged at physiological pH conditions, we optimized the neutral form parameters using two tests consisting of dose-dependent use-dependent block and use-dependent block, while preventing the remaining parameters from being modified during this step. Later, we fixed the neutral form parameters before starting a second phase optimization where charged flecainide variables were allowed to evolve. This phase studied the steady state availability, recovery from use-dependent block and the use-dependence of the drug’s IC_50_ for the sodium current at three frequencies of 0.2, 1 and 3 Hz, as in the work performed by Penniman et al. (2010), who used the PatchXpress system. In phase three, we ought to reproduce flecainide’s IC_50_ for I_NaL_ obtained by Matsukawa et al. (2019) with the latest recommended protocol by the Comprehensive *In-vitro* Proarrhythmia Assay (CiPA) initiative. Furthermore, we also studied the APD_90_ prolongation produced by 1.5 µM flecainide on the isolated endocardial cellular model, which we set to be as low as possible (target was 0%). We allowed charged flecainide affinities for the normal and bursting modes of the sodium current to be modified during the optimization. Parameters and equations of the final flecainide model are shown in Table 4. We performed a sensitivity analysis of flecainide’s parameters on the APD_90_ reduction of the isolated *in-silico* V411M mutated endocardial cells. The results of this analysis are included in the Supplementary Material (Supplementary Figure S5B) together with the definition of the parameters (Supplementary Table S3). Our study showed that the drug-induced attenuation of the APD_90_ prolongation caused by V411M was highly dependent on β4n and α4n (neutral flecainide trapping dynamics, modulated by p13 and p14). The affinity of charged flecainide for the bursting states also played a relevant role (modulated by p16). Finally, there was also a smaller yet noticeable dependence on an increase of β3+ and a decrease of α3+ (inactivation and recovery from inactivation of the channels bound to charged flecainide, modulated by p5 and p6).

**TABLE 4:** Transition rates of the optimized flecainide model.

Names	Equations
Transition rates	
α11+ and α11n	α11
α12+ and α12n	α12
β11+ and β11n	β11
β12+ and β12n	β12
αx+	9.72e-5×αx
βx+	2.85e-8×βx
α13+	1.29e-3×α13
α2+	2.93e3×α2
β3+	8.23e-9×β3
α3+	3.43e-7×α3
α4+	3.24×α2
β4+	3.27e-1×α3
αxn	1.54e-1×αx
α13n	2.49×α13
α2n	4.03e1×α2
β3n	7.07×β3
α4n	1.12e-3×α2
β4n	4.30e-3×α3
Affinities	
k_on = kc_on	drug_charged×diffusion
k_off = kc_off	2.15e1×(1e-6)×exp((−0.7×V×F)/(R×T))×diffusion
kb_on = kcb_on	k_on
kb_off = kcb_off	8.07e-1×(1e-6)×exp((−0.7×V×F)/(R×T))×diffusion
kn_on	drug_neutral×diffusion
kn_off	400×(1e-6)×diffusion
kni_on	kn_on
kni_off	5.4×(1e-6)×diffusion
knc_on	kn_on
knc_off	800×(1e-6)×diffusion
Diffusion	5500 M^−1 m−1^

The first column indicates transition rate names while the second column indicates their corresponding equations. The top section of the table defines drug transition rates, which are analogous to their respective wild type counterparts. The bottom section of the table defines drug-channel affinities. Nomenclature for transition rates and affinities: charged drug (+), neutral drug (n), closed states (c), inactivated states (i), bursting states (b), drug binding to the channel (on), drug unbinding from the channel (off).

**FIGURE 5:**
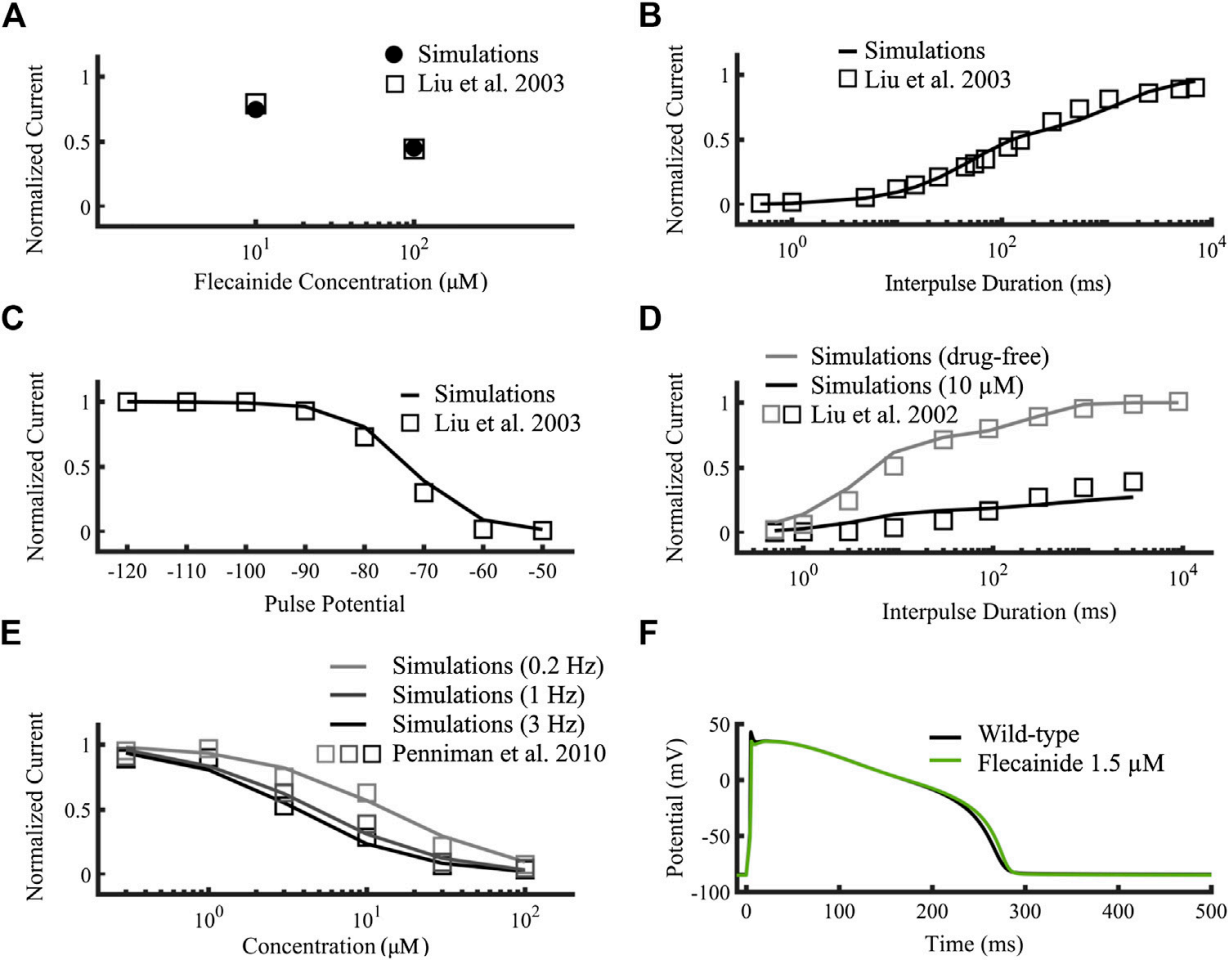
Optimization of the flecainide model. Targets were represented as open squares (Liu et al., 2002; Liu et al., 2003; Penniman et al., 2010) and simulations using our flecainide model as lines. **(A,B)** correspond to neutral flecainide, while the rest to neutral and charged flecainide. **(A)** Use-dependent block at 10 Hz of neutral flecainide. **(B)** Recovery from use-dependent block of neutral flecainide. **(C)** Steady state availability. **(D)** Recovery from use-dependent block (black). Wild-type drug free results (gray) were added for comparison. **(E)** Concentration-dependent availability at 3, 1 and 0.2 Hz (black, dark gray and light gray, respectively). **(F)** Simulated wild type isolated endocardial action potential time course at therapeutic concentrations (1.5 µM, green) compared to drug-free (black). I_NaL_ IC_50_ was 1.65 µM (not shown) and fitted to Matsukawa et al. (2019).

Figures 5A–D compare targets (open squares) obtained from experimental data and the simulated flecainide results (A: filled circles, B-D: black lines), namely, use-dependent and recovery from use-dependent block of neutral flecainide (Figures 5A,B), steady-state availability and recovery from use-dependent block, respectively. We included the wild type drug-free results in Figures 5D for comparison (gray symbols). Figures 5E compares the concentration-dependent availability of flecainide (lines) to experimental data from Penniman et al. (2010) (open squares) at 3, 1 and 0.2 Hz pacing rates (black, dark gray and light gray, respectively). The corresponding half-maximal inhibitory concentrations (IC_50_s) were 13, 4.73, and 3.59 µM, respectively. Figures 5F shows the simulated effects of 1.5 µM flecainide (green line) on the action potential of an isolated wild type endocardial cell, which causes an APD_90_ prolongation of 1.2% compared to drug-free conditions (black line). Details about the construction these curves were described in the Supplementary Material of this work. The simulated results obtained with our new flecainide model are in very good agreement with the experimental data. In addition, flecainide’s simulated IC_50_ I_NaL_ was 1.65 µM (not shown), very close to reference experimental data from Matsukawa et al. of 1.7 µM (Matsukawa et al., 2019).

#### Ranolazine

Ranolazine blocks hERG channels with an IC_50_ of approximately 35 µM (Moreno et al., 2013). In order to account for this effect, we reduced the I_Kr_ conductance by a 22.3% when applying 10 µM ranolazine, according to the Hill formalism (Hill coefficient of 1). Ranolazine optimization was performed by assessing I_Naf_ and I_NaL_ block dynamics, which were run in parallel in only one phase. Parameters and equations of the final ranolazine model are shown in Table 5. We performed a sensitivity analysis of ranolazine’s parameters on the APD_90_ reduction of the isolated *in-silico* V411M mutated endocardial cells. The results of this analysis are included in the Supplementary Material (Supplementary Figure S5C) together with the definition of the parameters (Supplementary Table S4). The analysis revealed that the drug-induced attenuation of the APD_90_ prolongation caused by V411M was highly dependent on an increase of β3+ and a decrease of α3+ (inactivation and recovery from inactivation of the channels bound to charged ranolazine, modulated by p5 and p6), as well as a decrease of βx+ and an increase of αx+ (recovery from slow inactivation and slow inactivation of the channels bound to charged ranolazine, modulated by p1 and p2). Although charged ranolazine’s affinities (modulated by p11 and p12) were not optimized during the fitting process, they were included in this analysis to make a comparison with flecainide results. They proved to be also key for drug-induced APD_90_ shortening and their role was even more relevant than in flecainide.

**TABLE 5:** Transition rates of the optimized ranolazine model.

Name	Values
Transition rates	
α11+ and α11n	α11
α12+ and α12n	α12
β11+ and β11n	β11
β12+ and β12n	β12
αx+	1.10e4×αx
βx+	1.27e-1×βx
α13+	2.7e-2×α13
α2+	1.15e5e3×α2
β3+	7.36e-2×β3
α3+	2.17e-2×α3
αxn	1.41e1×αx
α13n	3.41e2×α13
α2n	2.77e2×α2
β3n	2.87e-3×β3
Affinities	
k_on = kc_on	drug_charged×diffusion
k_off = kc_off	100.5×(1e-6)×exp((−0.7×V×F)/(R×T))×diffusion
kb_on = kcb_on	k_on
kb_off = kcb_off	1.5012×(1e-6)×exp((−0.7×V×F)/(R×T))×diffusion
kn_on	drug_neutral×diffusion
kn_off	400×(1e-6)×diffusion
kni_on	kn_on
kni_off	5.4×(1e-6)×diffusion
knc_on	kn_on
knc_off	800×(1e-6)×diffusion
Diffusion	5500 M^−1 m−1^

The first column indicates transition rate names while the second column indicates their corresponding equations. See caption of Table 3 for definitions of the abbreviations.

**FIGURE 6:**
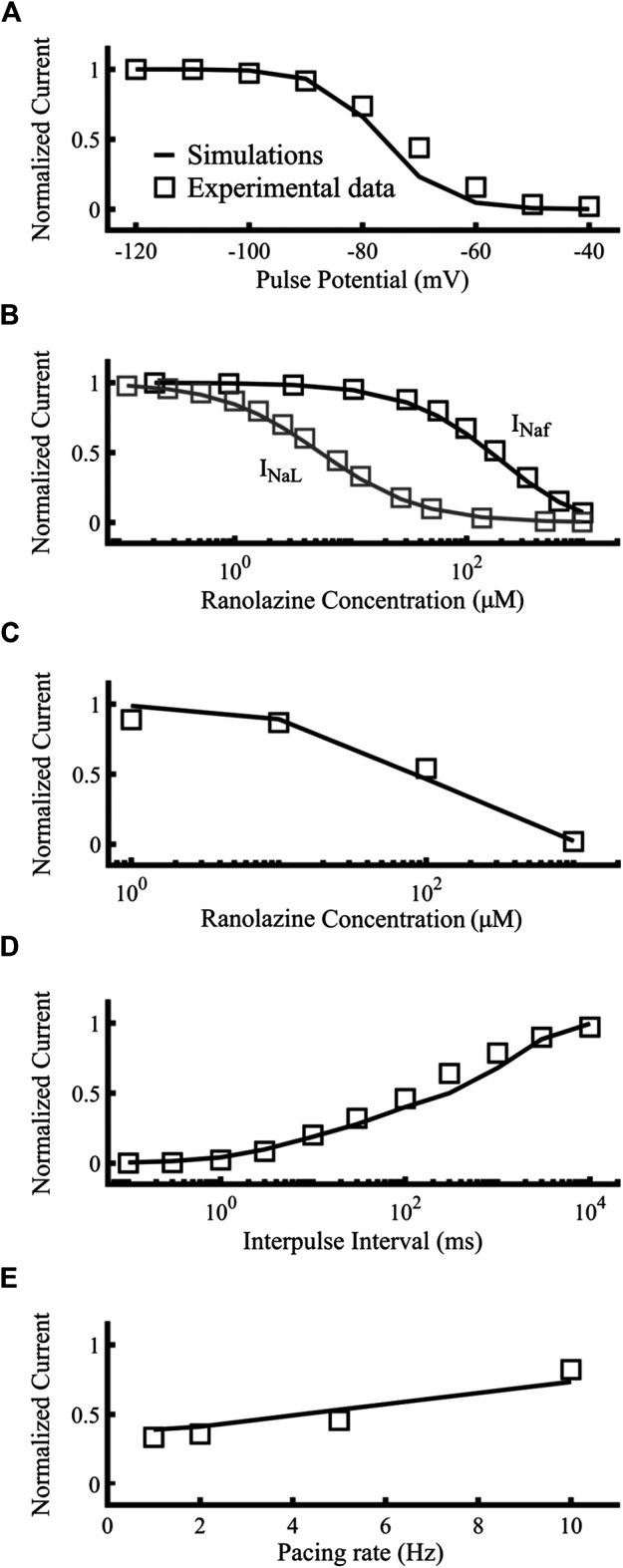
Optimization of the ranolazine model. Targets were represented as open squares and simulations with our new ranolazine model as lines. **(A)** Steady state availability of 10 µM ranolazine. **(B)** Tonic block of fast and late components of the sodium current. **(C)** Use-dependent block. **(D)** Recovery from use-dependent block of 10 µM ranolazine. **(E)** Frequency-dependent recovery from use-dependent block of 100 µM ranolazine. Experimental data are from Moreno et al. (2013).

Figures 6A–E compares the results targets (open squares) obtained from experimental data used by Moreno et al. (2013) to the simulated ranolazine results (black lines), namely, steady-state availability, tonic block of fast and late I_Na_, use-dependent block, recovery from use-dependent block and frequency-dependent recovery from use-dependent block, respectively. Protocols are described in Moreno et al. (2013) as well as the Supplementary Material. IC_50_s for I_Naf_ and I_NaL_ were 153.4 µM and 5.46 µM respectively, showing the specificity of the drug toward the latter. Overall, our ranolazine model was in agreement with the experimental data.

Effects of flecainide and ranolazine on the heterozygous SCN5A V411M phenotype.

Once the drug-free sodium currents and the drug-channel interactions were formulated, we proceeded to simulate the effects of ranolazine and flecainide in the presence of the V411M mutation on an isolated endocardial cell as well as on a ventricular transmural wedge.

**FIGURE 7:**
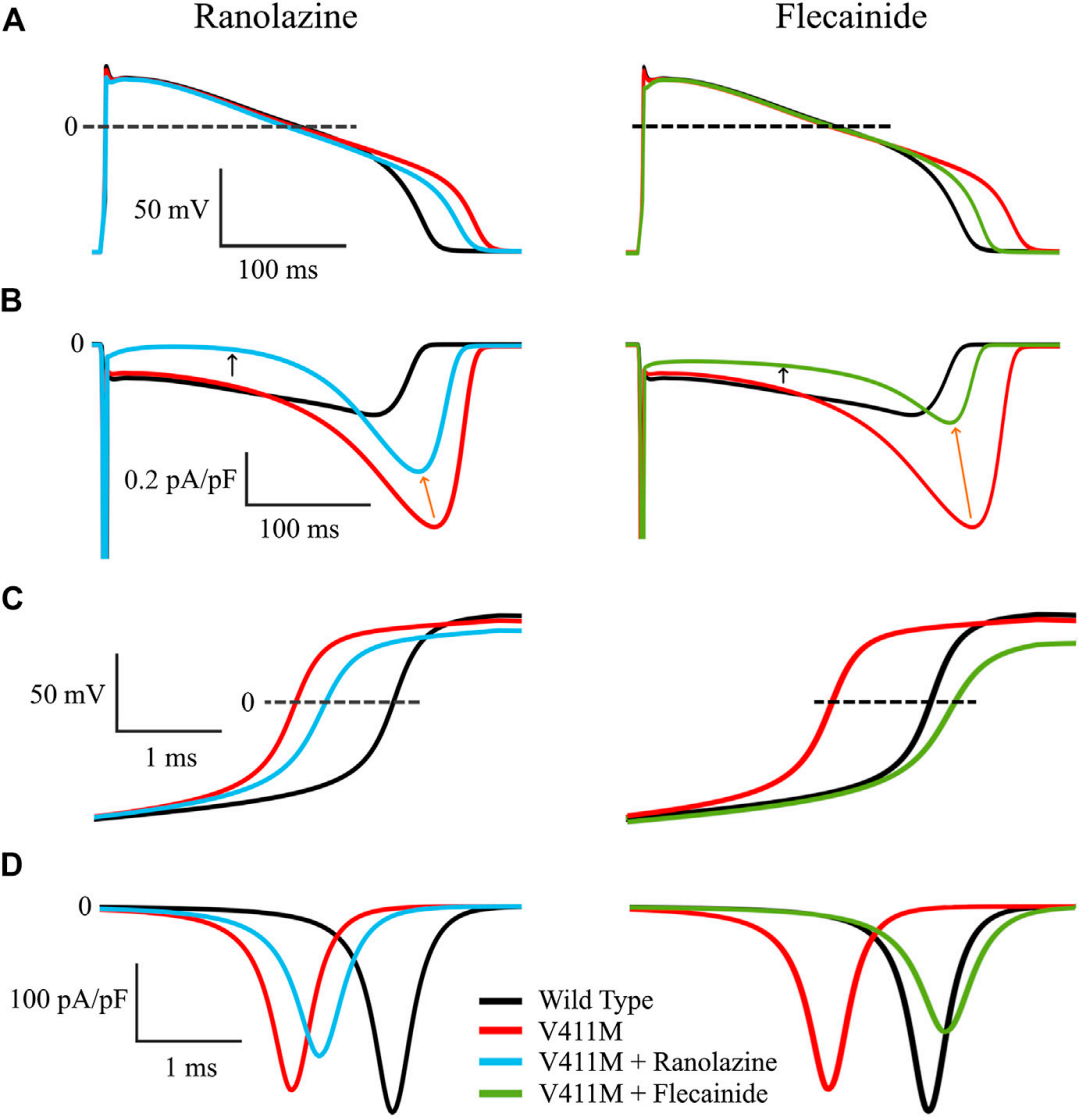
Simulated effects of 10 µM ranolazine (blue) and 1.5 µM flecainide (green) in isolated endocardial heterozygous SCN5A V411M mutated (red) cells. Drug-free wild-type (black) is also included for comparison. Action potential time courses **(A)**, I_NaL_ time course **(B)**, depolarization phase of the AP **(C)** and I_Naf_ time course **(D)**.

Figure 7 shows the effects of the application of therapeutic concentrations of flecainide (green) and ranolazine (blue) in isolated endocardial V411M mutated (red) cells, on the action potential time courses (Figures 7A), time course of the late component of the sodium current (Figures 7B), depolarization phase of the AP (Figures 7C) and fast component of the sodium current (Figures 7D). Therapeutic concentrations of ranolazine and flecainide produced a shortening of the heterozygous mutant APD_90_. Application of ranolazine resulted in a 298.1 ms APD_90_ (4.3% decrease) and exposure to flecainide resulted in a 284.5 ms APD_90_ (8.7% decrease), although the mechanisms involved in such results are different, as revealed by the corresponding I_NaL_ time courses (Figures 7B). In fact, ranolazine reduced the magnitude of the sodium current throughout the entire AP time course. Peak I_NaL_ current, measured as the maximum current during the repolarization, decreased from −0.75 pA/pF to −0.52 pA/pF (orange arrows), while plateau currents, measured at 100 ms from the start of the stimulus, decreased from −0.16 pA/pF to −0.018 pA/pF (black arrows). By contrast, flecainide had a major effect on I_NaL_ peak, whose value was much smaller (from −0.75 pA/pF to −0.32 pA/pF) while the effects during the plateau were less pronounced (from −0.16 pA/pF to −0.080 pA/pF). The effect of both drugs on the total amount of electronic charge carried by I_NaL,_ qNaL, which was calculated as the integral of I_NaL_ was also quantified and they turned out to be similar. Indeed, flecainide reduced qNaL from a value of −103.6 pC/pF in the mutant cells to −39.9 pC/pF and ranolazine produced a similar reduction to −49.2 pC/pF, both values being below the wild type value (−52.8 pC/pF). Figures 7C confirms the differences in the block of the fast component of the sodium current for both drugs. While ranolazine decreased the upstroke velocity from 259.9 V/s to 213.3 V/s, flecainide produced a stronger effect and reached 181.1 V/s as a consequence of its affinity for the fast component of the current. This is linked with the corresponding I_Naf_ time courses (Figures 7D). Ranolazine reduced I_Na_ peak from −251.6 pA/pF to −204.8 pA/pF while flecainide further reduced the peak to −172.6 pA/pF. Therefore, our isolated cellular simulations suggest that ranolazine could also be used to compensate the effects of the heterozygous V411M mutation.

**FIGURE 8:**
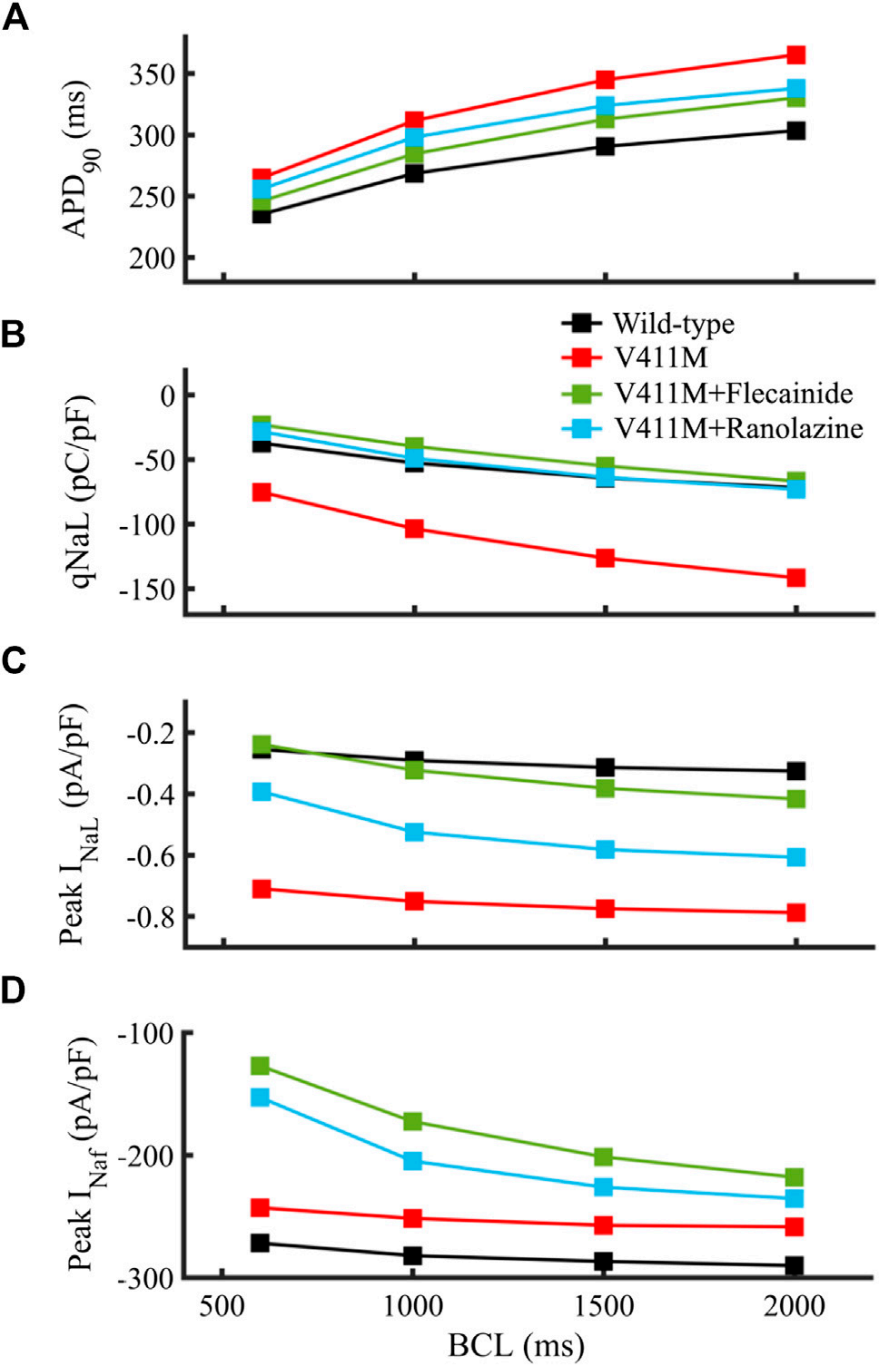
Simulated effects of 10 µM ranolazine (blue) and 1.5 µM flecainide (green) on the restitution dynamics of the APD_90_ **(A)**, qNaL **(B)** and peak I_NaL_ **(C)** in isolated endocardial heterozygous SCN5A V411M mutated (red) cells. Drug-free wild type (black) is also included for comparison. qNaL, the total amount of electronic charge carried by I_NaL_, was calculated as the area under the curve of I_NaL_.

We further examined the effects of applying therapeutic concentrations of flecainide and ranolazine in isolated endocardial heterozygous V411M mutant cells at fast and slow rates to see if the effects observed at 1 Hz hold at different frequencies. Figure 8 shows the effects of flecainide (green) and ranolazine (blue) on V411M mutated cells (red) at BCLs 600, 1,000, 1,500, and 2000 ms. This figure represents the APD_90_ (Figures 8A), the total amount of electronic charge carried by I_NaL_ qNaL (Figures 8B), and the peak values of I_NaL_ and I_Naf_ (Figures 8C,D). Our results show that both flecainide and ranolazine reduce the APD_90_ prolongation induced by the mutation for all the tested BCLs, flecainide’s APD_90_ reduction being higher than ranolazine’s (Figures 8A). The effects of these two drugs on qNaL are more pronounced than on APD_90_ as qNaL in mutant cells in the presence of the drugs is similar and even smaller than qNaL in WT cells (Figures 8B). The effects were similar at all BCLs although slightly greater at fast pacing rates (BCL 600 ms) compared to slow pacing rates (BCL 2000 ms). In addition, both flecainide and ranolazine were able to reduce peak I_NaL_ in the mutant phenotype at all pacing rates (Figures 8C). However, flecainide-induced reduction was more acute, peak I_NaL_ values being closer to the values in wild type. As a side effect, both drugs exacerbated the reduction of peak I_Naf_ caused by V411M mutation notably at fast rates, especially flecainide (Figures 8D). In fact, while flecainide reduced peak I_Naf_ to −127.1 pA/pF from a value of −242.9 pA/pF at a BCL of 600 ms, ranolazine reduced it to −152.8 pA/pF. Our study suggests that the therapeutic effects produced by ranolazine at 1 Hz in V411M mutated cells are maintained at fast and slow rates, as in the case of flecainide. Furthermore, ranolazine induced a smaller reduction of peak I_Naf_ than flecainide at all pacing rates.

Considering the mechanisms that define drug effectiveness, the sensitivity analyses we performed shed light into the key parameters (see Supplementary Figure S5). Firstly, they revealed that V411M mutation significantly increased I_Na_ activation and accelerated recovery from inactivation. This could explain a reactivation of the sodium current during repolarization due to an increased availability of sodium channels for activation earlier during the AP, which is supported by the fact that rates that control the fraction of channels in bursting mode (µ1 and µ2) were not altered. The main driver of flecainide’s effect on the mutation consisted of the trapping dynamics of the channels bound to neutral flecainide, but it also acted by binding to the bursting states and slightly reducing recovery from inactivation of the channels bound to charged flecainide (Supplementary Figure S9). By contrast, ranolazine depended on an even combination of decreased recovery from inactivation, increased slow inactivation and bursting state binding of the channels bound to charged ranolazine (Supplementary Figure S10). Ranolazine’s impact on bursting state binding was nonetheless greater than flecainide’s. In fact, when 10-fold ranolazine affinity reductions were considered, no drug-induced APD_90_ shortening could be observed, while in the case of flecainide it was 32% less effective compared to baseline flecainide (see Supplementary Figure S6). Likewise, ranolazine’s effect on the recovery from inactivation was more important. A 10-fold increase in β3+ doubled the reduction of the APD_90_ in ranolazine compared to an increase of a 9.7% in flecainide. Therefore, while both drugs shared a common affinity for the bursting states, their resulting qNaL, peak I_NaL_ and APD_90_ reductions relied on different mechanisms, indicating that flecainide took advantage of its trapping dynamics while ranolazine relied on a combination of factors including its high affinity for the bursting mode.

**FIGURE 9:**
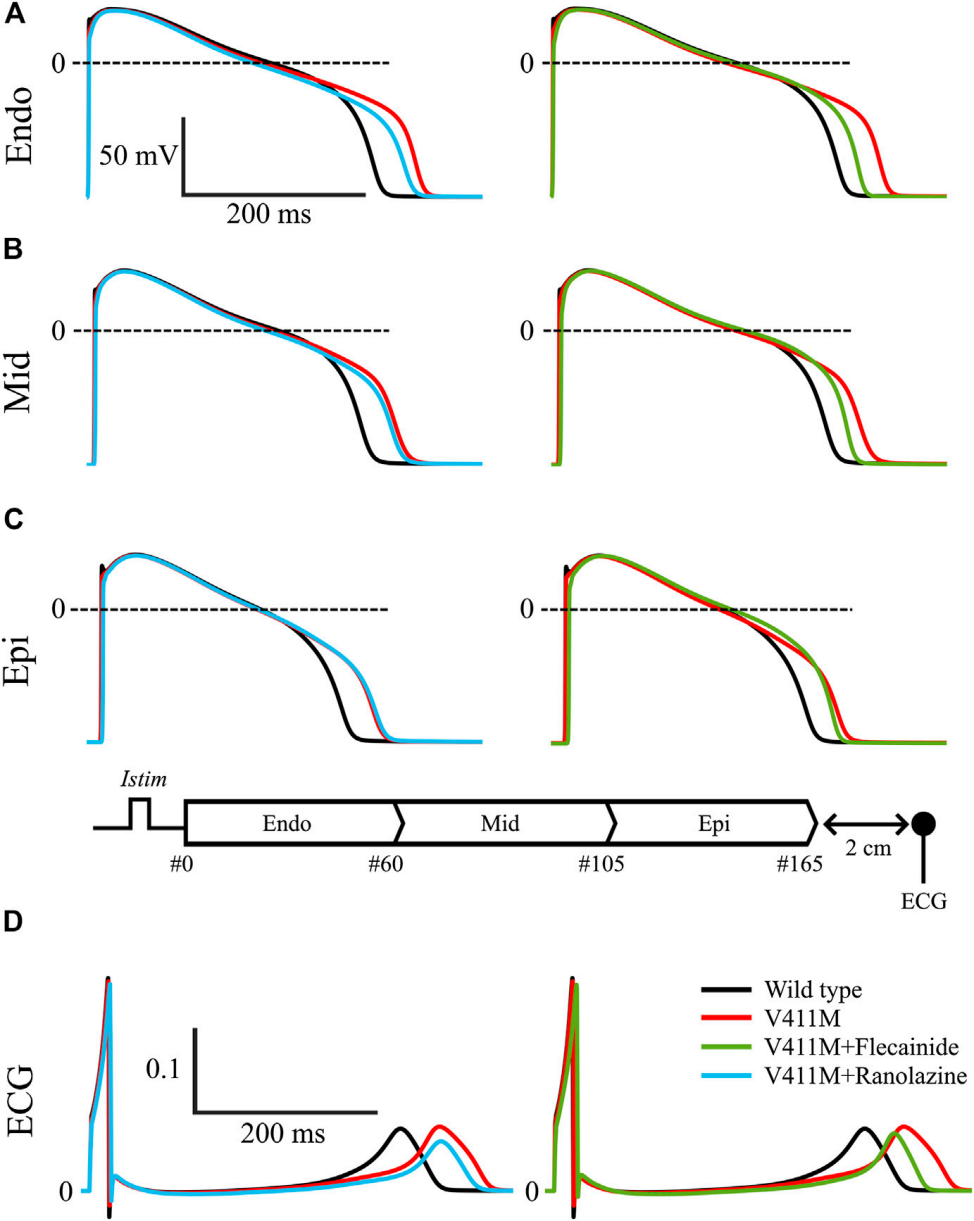
Simulated effects of 10 µM ranolazine (blue) and 1.5 µM flecainide (green) on action potentials and pseudo-ECGs in the presence of SCN5A V411M heterozygous mutation (red) using a one-dimensional (1D) tissue model of a transmural wedge preparation. Drug-free wild type (black) was also included for comparison. Action potentials from endocardial **(A)**, midmyocardial **(B)** and epicardial cells **(C)**. Representation of the simulated transmural 1D tissue, and its corresponding pseudo-ECG **(D)** obtained with a virtual electrode 2 cm away from the last epicardial cell.

Next, we simulated the effects of ranolazine and flecainide on the transmural wedge model. Figure 9 shows the simulated effects of ranolazine (blue) and flecainide (green) on APs and pseudo-ECGs in the presence of V411M heterozygous mutation using a one-dimensional (1D) tissue model of a transmural wedge preparation. This figure also includes the results in drug-free wild type (black) conditions for comparison. Figures 9A–C represent the APs of selected cells from the strand, specifically, #16, #80 and #150. For the sake of clarity, we included a diagram of the simulated strand. Figures 9D contains calculated pseudo-ECG time courses with a virtual electrode 2 cm away from the last epicardial cell. As shown in Figures 9A–C, APD decreased from endocardial to epicardial cells for the simulated wild type drug free transmural wedge preparation, as the progressive left shift of the repolarization of the selected action potential time courses indicates. APD_90_ values were 328.8, 310.7, and 279.6 ms for the selected endocardial, midmyocardial and epicardial cells, respectively. The simulated QT segment lasted 336.8 ms and the QRS 18.4 ms. The APD_90_ prolongations produced by the V411M heterozygous mutation were greater for endocardial cells, followed by midmyocardial and epicardial cells, whose APD_90_ were 377.1, 350.7, and 314.5 ms respectively, corresponding to a prolongation of 14.7, 12.9, and 12.5% compared to WT. The ECG showed a prolongation of the QT segment of 48 ms (14.3%), which is close to clinical observations (Horne et al., 2011; Carrasco et al., 2012). Therapeutic concentrations of ranolazine (10 µM) in the presence of the V411M heterozygous mutation decreased the APDs of endocardial (363.9 ms, −3.5%) and midmyocardial (343.8 ms, −2.0%) cells. The calculated QT segment also decreased to 372.4 ms (−3.2% compared to heterozygous mutation) in the presence of this drug. Interestingly, the QRS values slightly increased (10.9%) as expected from the small interaction of ranolazine with the fast component of the sodium current. Flecainide therapeutic concentrations (1.5 µM) caused a reduction of the APD_90_s in all the selected cells. Specifically, endocardial, midmyocardial and epicardial APD_90_s were 350.2 ms (−7.2%), 330.3 ms (−5.8%), and 303.5 ms (−3.5%), respectively. Flecainide reduced the QT segment to 357.1 ms (−7.2%). The effects of the drug on I_Naf_ were stronger than ranolazine’s, as expected, widening the QRS complex by a 23.4%. Interestingly, the results obtained using the isolated endocardial model translated very well to the strand model. Specifically, both drugs reduced the QT segment duration by a similar extent, although flecainide was slightly more effective, and widened the QRS more than ranolazine, which is related to the corresponding reductions in upstroke velocity. Therefore, our transmural wedge simulations reinforce that ranolazine could also be used to compensate the effects of the V411M heterozygous mutation.

**FIGURE 10:**
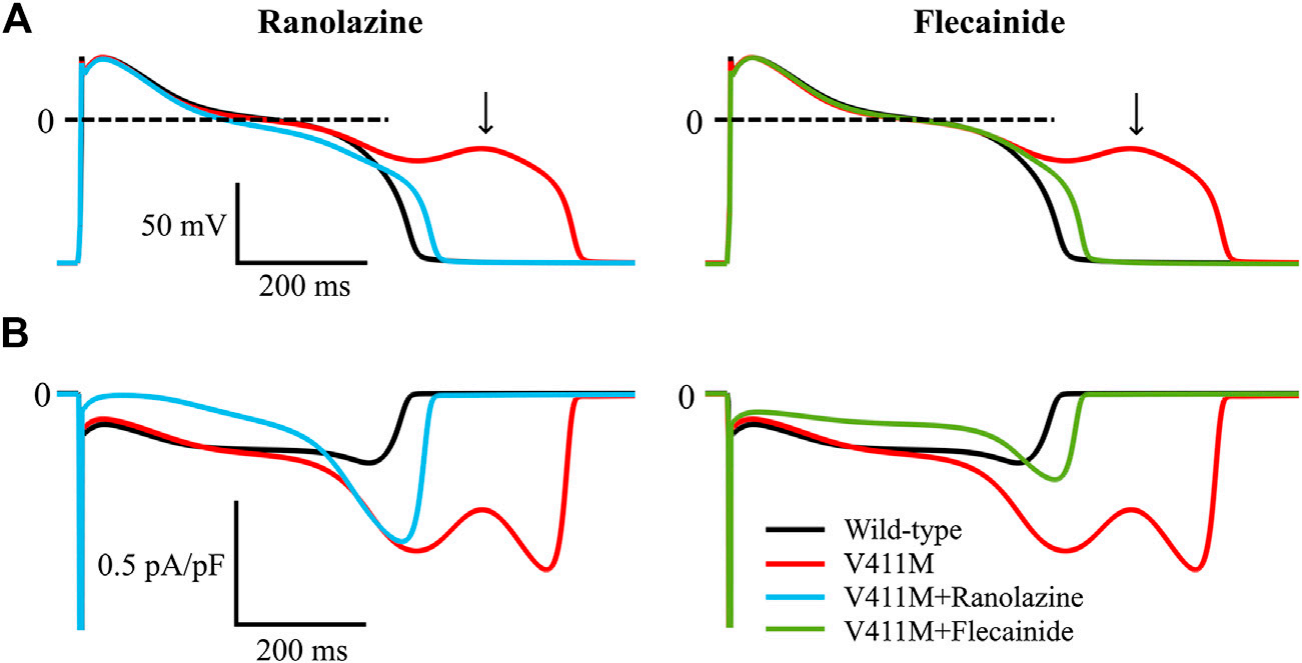
Simulated effects of 10 µM ranolazine (blue) and 1.5 µM flecainide (green) on EAD generation in slow paced (BCL = 3,000 ms) isolated midmyocardial heterozygous SCN5A V411M mutated (red) cells. Drug-free wild type (black) is also included for comparison.

As arrhythmias associated to LQT3 typically manifest at bradycardic rates, we investigated the potential of ranolazine to mitigate abnormal electrophysiological phenotypes arising from the V411M mutation at slow pacing rates. Figure 10 illustrates the simulated steady-state time courses of membrane potential (A) and I_Na_ (B) of an isolated midmyocardial heterozygous V411M mutant cell paced at a BCL of 3,000 ms in the absence of any drug (red) and in the presence of therapeutic concentrations of flecainide (green) and ranolazine (blue). Wild type time courses (black) were also included for comparison. The mutation increased I_NaL_ during the late plateau (red trace), which led to EADs (black arrow). Therapeutic concentrations of both flecainide and ranolazine reduced I_NaL_ and successfully normalized AP morphology and avoided EAD generation. These results suggest that ranolazine could also prevent the development of potentially arrhythmia-triggering EADs at slow pacing rates in patients with heterozygous V411M, similarly to flecainide.

## Discussion

### Main Findings

In this work, we aimed to study the potential use of ranolazine as a treatment for patients with LQT3 caused by the heterozygous V411M mutation, and to compare its effects to those produced by flecainide, using computational simulations. For this purpose, first, we optimized existing Markovian models of the wild type cardiac sodium current in the absence of drugs and under exposure to flecainide and ranolazine, which are two sodium current-blocking antiarrhythmic drugs. The proposed wild type model improves the I_NaL_ time course, the current-voltage relationship and the endocardial APD_90_ restitution curve. The models of ranolazine and flecainide were adapted to the new wild type, and in the case of flecainide, to reproduce new experimental and clinical data. Indeed, flecainide optimization was constrained with new tests assessing its I_NaL_ blocking potency using the latest recommended patch-clamp protocol, and the prolongation of the APD_90_ of the endocardial cellular model under exposure to therapeutic concentrations. Then, we generated a formulation of the gain-of-function V411M mutation to reproduce voltage clamp recordings and the QT prolongation clinically observed. Simulation of the electrophysiological activity in the presence of this mutation showed an increase of I_NaL_ during late repolarization. Additionally, we tested the effectiveness of both drugs as treatments for the mutation in an isolated cellular and a transmural wedge model and we found that they were both capable of countering APD_90_ and QT prolongations, although the subjacent electrophysiological mechanism differed. Indeed, while ranolazine reduced I_NaL_ throughout the action potential, flecainide was more specific of the late repolarization peak at a wide range of pacing rates. Additionally, EADs triggered during slow pacing were prevented by therapeutic concentrations of flecainide and ranolazine alike.

Therefore, our study suggests that ranolazine could be applied in patients with LQT3 caused by the heterozygous V411M mutation that could not be treated with flecainide.

### Ranolazine and Flecainide for LQT3 Patients

Several authors have shown that ranolazine could reverse abnormalities found in patients with LQTS type 3 characterized by an abnormal increase in I_NaL_ (Kaufman, 2008; Moss et al., 2008; Chorin et al., 2016), which is in accordance with our results. Moreno et al. (2013) simulated the effects of ranolazine in the presence of the SCN5A ΔKPQ mutation associated to a severe overlapping phenotype including LQTS type 3, Brugada syndrome and conduction disease (Moss et al., 2005). Moreno and coworkers concluded that a reduction of I_NaL_ with ranolazine might be an effective therapeutic strategy. Indeed, this mutation induced an increase in I_NaL_ that causes a LQTS type 3 where patients frequently suffered from tachyarrhythmias. The authors found that ranolazine was able to stabilize the mutation induced EADs by considerably reducing I_NaL_ in three human cardiomyocyte isolated cell action potential models, reporting a 50% or higher reduction of the APD_90_ at 10 µM ranolazine. In our work, ranolazine reduced by a 4.3% the APD_90_ in isolated endocardial cells with the SCN5A V411M mutation. The lesser extent of the reduction observed in our simulations compared to that of Moreno et al. (2013) can be due to the fact that ranolazine binds with high affinity to bursting channels, whose proportion is much higher in the ΔKPQ mutation than in the V411M mutation. Specifically, the ΔKPQ mutation provoked up to a five-fold increase of the persistent I_NaL_ by drastically increasing the proportion of channels in bursting mode (Moreno et al., 2013), opposite to the V411M mutation. Therefore, our simulation results reveal that ranolazine could be useful in the treatment of patients with the SCN5A V411M mutation, although its beneficial effects may not be as dramatic as in the case of the ΔKPQ mutation.

Regarding flecainide, it was able to almost completely compensate the mutation phenotype in combination with β-blocker (propranolol) in two case-reports (Carrasco et al., 2012; Blich et al., 2019). In our work, flecainide reduced the QT interval of the isolated endocardial cell to a point comprised between the wild type values and those corresponding to the mutated cells, suggesting a smaller effect that in the clinical practice. Possible reasons for the discrepancy in the APD reductions could consist of *in-silico* and *in vitro* differences, the drug I_Kr_ or I_NaL_ real blocking potency or in other ionic current densities that obviously interact in the human model and are obviated in the transfected cell models. The real blocking effect of flecainide on INaL remains elusive. Although we used updated IC_50_ data, we acknowledge that IC_50_ values reported in the literature widely range from 3.4 µM (Belardinelli et al., 2013) to 44 µM (Nagatomo et al., 2000), with medium potencies observed such as 19 µM (Crumb et al., 2016). More recent studies aimed at determining I_NaL_ IC_50_ using a novel protocol for high throughput ion channel screening showed much lower values, such as 1.7 µM (Matsukawa et al., 2019) or 1.9 µM (Guo and Jenkinson, 2019). These protocols give more accurate values of the INaL blocking power of the drug during the action potential since they measure the current elicited by a decreasing ramp after a pulse that is intended to mimic the repolarization phase of the action potential, contrary to previous studies that measure the remaining I_NaL_ current at a fixed interval after application of a voltage step (Guo and Jenkinson, 2019). Our optimized flecainide model used these new protocols and data, representing an improvement over the original Moreno et al. (2013) model (Moreno et al., 2013). Finally, even though flecainide may produce a small QTc prolongation (8%^3^) in clinical practice, QRS widening was the main contributor (60–90%^3^), an effect that is related to I_Naf_ block. These clinical observations are in keeping with the absence of APD_90_ prolongation in ventricular cells (Smallwood et al., 1989; Lu et al., 2008), which we took into consideration in our fittings. Despite the I_NaL_ IC_50_ test showing good results in our work, flecainide incompletely counteracted the APD_90_ prolongation produced by the presence of the heterozygous mutation. A possible reason could be that we had overestimated the IKr blocking effect of flecainide due to *in vitro-in vivo* differences in terms either of drug exposure or the values of the ionic current conductances in the action potential model, whose experimental measurement is subject to a high variability. Indeed, a smaller contribution of I_Kr_ to the action potential time course would result in a smaller impact on the repolarization reserve of the cell and a subsequent shorter APD_90_ prolongation, thus helping close the gap between clinical observations and the simulations with flecainide in the presence of the mutation. To assess the impact of blocking I_Kr_ on APD_90_ prolongation we simulated the effects that therapeutic concentrations of flecainide and ranolazine would have on isolated heterozygous mutant cells without considering the effects on I_Kr_ and I_K1_. Our results show that both drugs would reduce the APD_90_s of the mutant cells close to, and even below, the WT value (Supplementary Figure S6).

In conclusion, our simulation results show that ranolazine induces a similar qNaL reduction to flecainide, bringing it close to wild type values, and reduces QT/APD90 prolongation caused by V411M mutation, although to a lesser extent than flecainide. Like flecainide, ranolazine was also able to prevent arrhythmia triggers, suggesting that it could be useful in treating LQTS type 3 patients carrying V411M mutation. Unlike flecainide, ranolazine would also be safe in the presence of structural heart disease (Andrikopoulos, 2015) and the scarcity of evidence also supports its use in patients with overlap phenotype manifesting with Brugada syndrome or conduction disease in addition to the LQTS type 3 phenotype, although such a behavior has never been reported for V411M carriers.

### Ionic Models

The crucial role of I_NaL_ in cardiac electrophysiology puts into perspective some relevant contributions of this work, namely, the improvement of the formulation of the sodium current and the updates of the flecainide and ranolazine models. In the present work, we improved I_NaL_ by increasing its contribution to the APD restitution and optimizing its time course. This resulted in a reduction of the late peak (compared to Moreno et al., 2013) and an increment over the whole action potential. According to Horne et al. (2011), the V411M mutation induced an increment of the sodium current during repolarization. They suggested that the cause was an increase in window current due to reactivation of the channels. Our work is in agreement with the authors as our simulations reproduce the shifts of the activation and inactivation steady state curves, resulting in a greater window current that occurs during repolarization. Horne et al. (2011) suggested that the specific open state blocker flecainide would be useful in treating the condition, which was later confirmed by Carrasco et al. (2012). Our work provided a mechanistic insight into why flecainide is a good treatment by showing its specific effect on the V411M mutation induced I_NaL_ peak and tested ranolazine as a possible alternative treatment.

### Limitations

Our optimizations were designed using the Nelder-Mead algorithm, as in other studies (Moreno et al., 2011; Moreno et al., 2013; Moreno et al., 2016; Romero et al., 2015; Yang et al., 2016b). This algorithm benefits from being simple to implement, parallelizable and requiring “less” computational power than other optimization algorithms. However, some notable limitations include its high likelihood of finding a local instead of a global minimum and its slowness, requiring a higher number of iterations (Moreno et al., 2016). As our goal was to refit the existing models by adding a few new tests, we limited the number of iterations to 300, which reduced the computational cost of our optimizations. In order to reduce the likelihood of finding a local minimum we randomized 10 sets of initial parameters with a 10% variability instead of creating radically different sets of parameters, as the initial parameters already yielded close results to many of the tests.

Simulations of the APD_90_ restitution curve in wild type, the prolongation of the APD_90_ for V411M mutated cells and the effects of flecainide on APD_90_ for each iteration were not obtained by applying a train of pulses until reaching the steady state, as it would have resulted in a very high computational demand. As explained in Methods, we first paced the drug-free wild type model for 300 s at 1 Hz pacing rate for each case and the final states were saved. They were used as initial states in the optimization procedure and a train of 40 pulses was applied to the isolated endocardial cell models for each iteration. Additionally, we brought the isolated endocardial, midmyocardial and epicardial models to steady state with 300 pulses at 1 Hz pacing rate to prepare for tissue simulations.

Horne et al. analyzed the V411M mutated sodium channels with a patch-clamp protocol including a voltage ramp which they used to assess I_NaL_ current density Horne et al. (2011), (Figure 5). The increase in I_NaL_ that was required to reproduce clinical QT prolongations in our model was greater than the one registered by Horne et al. *in-vitro*. Therefore, we did not include this protocol in our optimization procedure. A possible explanation to this discrepancy could be that the current density changes that the mutation produces in the transfected HEK293 cells may differ from those in human cardiomyocytes. In the heart, Nav1.5 is associated not only with several accessory proteins that modulate its trafficking and biophysical properties, but also with other structural and signaling complexes in the cardiomyocyte, such as caveolin and syntrophin (Bohnen et al., 2017). Nevertheless, as new works make their way to the literature, their data could be integrated to this model to improve it further favored by its inherited versatility.

A recent work by Horváth et al. (2020) compared human, guinea pig and dog I_NaL_ time courses using the same protocols. They found that dog and human I_NaL_ showed a similar “decrescendo” behavior, contrary to guinea pig I_NaL_ whose time course followed a “crescendo” pattern in addition to having slower inactivation dynamics. They concluded that dog cardiomyocytes were more suitable for I_NaL_ pharmacological studies. Although we used a guinea pig time course in the fitting of our wild-type model (Horvath et al., 2013), we still improved the formulation proposed by Moreno et al., 2013 as we reduced the contribution of the current to the late repolarization phase. Our improved model shows now a flatter time course similar to the one in the ORd modified by Dutta and coworkers (Dutta et al., 2017) at 1 and 2 Hz.

Since we did not account for β-stimulation in our models, we used the QTc values of our patients while being treated with β-blocking drugs to model the effects of the V411M mutation. However, the reference values were obtained from subjects lacking such treatment.

A recent work by Zhu et al. (2019) found that sodium channel block by mexiletine differed between LQTS type 3 induced mutations. In this work, the same values of drug affinities were used for wild type and V411M mutated cells, although they could be altered by the mutation (Ficker et al., 1998). In order to take into account a possible difference in drug affinities between wild type and V411M mutated cells additional experimental data would be needed.

Our study would also benefit from the simulation of flecainide and ranolazine using a population of models that account for natural variability among individuals of the same species (Sobie, 2009; Britton et al., 2013). This would help to better predict the effectiveness in population of patients.

Finally, it should be noted that our results might be model or parameter dependent, although qualitatively they should hold. The models used in our work were the result of fitting the parameters of the original Moreno and coworkers Markov formulation of I_Na_ to reproduce experimental results from the literature using a specific action potential model. This was the Grandi et al. (2010) with Soltis and Saucerman (2010) model, which has already been used in similar conditions than our work (Moreno et al., 2013). Inserting our I_Na_ formulation in a different action potential model would require refitting of the parameters to reproduce experimental data. Likewise, the parameters we obtained result from the particular dataset we used to fit them, which covered a wide range of I_Na_ dynamics in all optimizations. Therefore, the assessment of ranolazine’s and flecainide’s impact on the V411M mutation phenotype using different action potential models or datasets might be quantitatively different, but qualitatively similar, as the models should capture the mechanisms of both mutation and drugs.

All in all, we believe that these limitations do not jeopardize the main conclusions of this work.

## Conclusion

In this work, we improved an existing Markovian wild type cardiac sodium channel model with new I_NaL_ experimental data. Then, we fitted a new model for the gain-of-function V411M mutation. We also updated two antiarrhythmic drug models—flecainide and ranolazine—that we used to test their efficacy as treatments for the V411M phenotype. We found that both drugs could be beneficial because of their APD_90_ and QT shortening effect at a wide range of pacing rates. Both reduced the total charge carried by I_NaL_ close to wild type values, but flecainide was more effective at reducing the mutation induced late sodium current peak. Ranolazine reduced peak I_Naf_ to a lesser extent than flecainide, which would be beneficial for some patients. Importantly, our simulations suggest that, similarly to flecainide, ranolazine has the potential to prevent arrhythmia triggers during bradycardia episodes in patients with V411M mutation.

Our study is the first to model the V411M mutation in great detail, providing a deeper understanding of the underlying mechanisms of the mutation phenotype. We also provided insights into the mechanisms that drive the effectiveness of two antiarrhythmic drugs, flecainide and ranolazine. Our results support the fact that, while flecainide was better suited to counter the mutation, ranolazine could be beneficial via a different mechanism. Therefore, our computational study suggests that patients with LQTS 3 caused by the heterozygous V411M mutation could be treated with ranolazine. Finally, our work provides new tools to further explore new treatments for the V411M or other similar mutations.

## Data Availability Statement

The raw data supporting the conclusions of this article will be made available by the authors, without undue reservation, to any qualified researcher.

## Ethics Statement

The studies involving human participants were reviewed and approved by Comité Ético de Investigación Biomédica, Hospital Universitario y Politécnico La Fe, Valencia (Spain). Written informed consent to participate in this study was provided by the participants' legal guardian/next of kin.

## Author Contributions

JC, LR, BT, and JS contributed to the conception and design of the study; MA, EZ, AM, and SP obtained and analyzed the clinical data; JC wrote the code, performed the optimizations, created the figures and tables and wrote the first draft of the manuscript; JC, LR, and EZ wrote sections of the manuscript. All authors contributed to manuscript revision, read and approved the submitted version.

## Funding

This work was partially supported by Fondo Europeo de Desarrollo Regional (FEDER, “Unión Europea, Una forma de hacer Europa”) with the Ministerio de Economía y Competitividad (DPI2015-69125-R), Dirección General de Política Científica de la Generalitat Valenciana (PROMETEO/2020/043) and Instituto de Salud Carlos III (La Fe Biobank PT17/0015/0043), as well as by Vicerrectorado de Investigación, Innovación y Transferencia de la Universitat Politècnica de València with Ayuda a Primeros Proyectos de Investigación (PAID-06-18), and by Memorial Nacho Barberá.

## Conflict of Interest

The authors declare that the research was conducted in the absence of any commercial or financial relationships that could be construed as a potential conflict of interest.

## Footnotes

1
www.QTcalculator.org[accessed 2019]

2 U.S. National Library of Medicine, Dailymed: https://dailymed.nlm.nih.gov/dailymed/ [accessed 2019]

3 U.S. National Library of Medicine, Dailymed: https://dailymed.nlm.nih.gov/dailymed/ [accessed 2019]
