# Supplementary material for: Ranolazine as an Alternative Therapy to Flecainide for SCN5A V411M Long QT Syndrome Type 3 Patients
Source: Front Pharmacol. 2020 Dec 17;11:580481. doi: 10.3389/fphar.2020.580481 (PMC7845660; doi:10.3389/fphar.2020.580481)
Supplement: Supplementary file 1 [file datasheet1.pdf]

Supplementary Material

1 Expanded materials and methods

1.1 In silico patch-clamp protocols

To study the sodium channel dynamics and reproduce the experimental results, we reproduced the described conditions (pH, temperature and sodium concentrations) while integrating the sodium current over the described time and voltage protocols. Temperature derived changes were applied with a scaling factor calculated with a Q_{10} of 3 as follows.

$$T_f = \frac{1}{Q_{10}^{\frac{37-T}{10}}}$$

Where T represents the temperature (in Celsius).

Patch clamp simulations were usually performed at room temperature (according to the reference experimental protocol), while action potential simulations were always carried out at 37ºC.

Both extracellular and intracellular sodium concentrations were fixed during in silico patch-clamp experiments, while intracellular sodium concentrations were dynamically updated in action potential cellular models. Results were also extracted following the methods described in their respective references. These include indications concerning when and where to measure the current as well as how to normalize the data if needed. Experimental data points were manually digitized from their original figures.

Patch-clamp protocols were simulated using custom Matlab2014b (The Mathworks, inc) code including an ordinary differential equation (ODE) solver with variable time step limited to a maximum of 0.5 ms.

1.2 General Optimization Protocols

We used the function “fminsrchbnd” (Moreno et al., 2011, 2013, 2016), which is an implementation of the Nelder-Mead simplex that allows boundaries to be set to the parameters that ought to be fitted.

Termination conditions were met when a maximum number of iterations - or cost function evaluations - was reached (300). This could also happen when error variation between consecutive iterations stayed below a tolerance level of 0.01, but that rarely was the case due to the limited number of iterations.

Scores for each test were computed as the sum of squared differences between test results and reference data. Dividing the score by the test’s number of samples was done to normalize the contribution of each test. Nonetheless, to compare scores from very different units (i.e.: milliseconds and normalized values), custom scalar factors were added to each test’s score. The final error score of a cost function was determined by the sum of the scores of every single test in that function.

Optimizations were prone to being unable to surpass some local minima rather than finding the best solution (Moreno et al., 2016). Specifically, due to the models being already fit to several of the tests, the models were not able to “exit” the current local minimum. Therefore, we gave each optimization a randomly generated vector of initial variables (10% variability) and launched several optimizations for every model. To ensure reproducibility, we controlled the initial vectors by giving the random number generator a seed to start with.

1.3 Optimization Functions

1.3.1 Optimization of the Wild-Type Sodium Current Model

The optimization of the Wild-Type sodium channel contains the following tests of which six were extracted from Moreno et al. 2011 (Moreno et al., 2011) including steady state availability (SSA), activation (ACT), recovery from inactivation (RFI), recovery from use-dependent block (RUDB) and time to 50% activation (Tau50). Three new tests were added running cellular simulations to build the APD_{90} Restitution Curve (REST), optimize the slow sodium current IV (I_{NaL} IV) and time course (I_{NaL}) according to AP clamp recordings (Horvath et al., 2013; Hegyi et al., 2018). The model was also constrained by maximum upstroke velocity (max dV/dt) and channel Mean Opening Time (MOT).

Before starting the optimization procedures, we stabilized the cellular model by running a 300-second train of pulses at several Basic Cycle Lengths or BCLs (300, 400, 500, 1000, 1500 and 2000 ms). These simulations served as steady state starting points for I_{NaL} and REST tests.

Every iteration, we first stabilized the I_{Na} Markovian model by applying a single pulse from -100 mV to -10 mV during 200 ms, followed by a 5-s, -100 mV, resting membrane potential phase. We used the final states from the latter as a starting point for every test involving patch-clamp protocols.

I_{NaL} time course: We applied a train of 40 stimuli at 1 Hz to the modified isolated endocardial model and saved the last beat for analysis. We extracted three parameters from the I_{NaL} time course as can be observed in *Supplementary Figure S1.* Firstly, we defined the “dome” as the maximum late sodium current (registered after the fast sodium current had inactivated). Secondly, we defined the “valley” as the minimum current between peak I_{Na} fast and the dome. Finally, we defined the half-time taken by the current to reach the dome from the valley as $t_{1/2}$. Both valley and $t_{1/2}$ were normalized to the time and current of the dome. We set reference dome current to -0.34 pA/pF (Dutta et al., 2017) , a dome-to-valley value to 0.59 and $t_{1/2}$ value to 0.63 (Horvath et al., 2013).

Steady state availability: We applied a single 25 ms pulse to -10 mV from a 5-second variable potential test pulse from -120 mV to -40 mV (5 mV intervals), extracted peak currents elicited by the second pulse, normalized them to tonic block at -120 mV and plotted them against pulse voltage.

Activation: We applied a 25 ms test pulse to variable potentials from -80 mV to 20 mV from a resting potential of -100mV. Then, we calculated the resulting channel conductance from the elicited peak currents, normalized the results to maximum conductance and plotted them against pulse potential. Conductance values for test potentials above the potential of maximum conductance were set to 1.

Recovery from inactivation: We evaluated recovery from inactivation with a standard double-pulse protocol from -100mV to -10mV. The second pulse was delayed with increasingly higher time intervals ranging from 0.1ms to 6s. We extracted peak currents elicited by the second pulse and normalized them to peak currents elicited during the first pulse, then represented the data against time intervals.

Recovery from use-dependent block: First, we simulated a train of 300, 25-ms pulses, from -100 mV to -10 mV, at a pacing rate of 25Hz. Potential was set back to -100mV before a last pulse to -10 mV was applied after a variable delay ranging from 0.5 ms to 9 s. We extracted maximum current peaks elicited during the second pulse and normalized them to their maximum value. We represented the data against time intervals.

Tau 50% activation: Following the Activation protocols, we measured Tau50 as the time the current took to reach 50% of the maximum peak current at the beginning of each pulse.

APD_{90} restitution curve: We performed 40-second simulations with the modified endocardial model at 300, 400, 500, 1000, 1500 and 2000 ms BCLs. We carried every simulation in parallel and saved the last beat for further analysis. We extracted the APD_{90} of saved beats by calculating the interval between de time of max dV/dt and the time of 90% repolarization. We plotted the data against BCL and compared the results to the ORd model's reference (O'Hara et al., 2011).

Maximum upstroke velocity was extracted during BCL 1000 ms simulations. Target value was set to 250 V/s.

MOT was calculated as in Moreno et al. 2011 (Moreno et al., 2011).

1.3.2 Optimization of the SCN5A V411M Mutation Model

Optimization of the SCN5A V411M mutation model included protocols testing the following dynamics, namely, activation (ACT), inactivation (INACT), inactivation time constants (TauINACT), current-voltage relationship (IV) and prolongation (Prol). Except the latter two, all protocols follow the same methods as in Horne et al. 2011.

Before the optimization, we paced the single cell model with a train of 300 square stimulus at 1 Hz to reach steady state.

We ran one iteration of the cost function before starting the optimization in order to create a starting set of curves corresponding to the wild type model. Target values were generated and applying the relative wild-type-to-mutation changes in current dynamics observed by Horne et al. (Horne et al., 2011).

Activation: We applied a 200-ms pulse to a variable potential from -80 mV to 30 mV and extracted the resulting maximum conductances from the elicited peak currents, which we normalized to the highest value. We represented the data against pulse potential and fitted it to the following Boltzmann equation:

$$\frac{G}{G_{max}} = \frac{1}{1 + e^{\frac{V_h - V}{s}}}$$

Where Vh is the half-maximal voltage and s is the slope of the curve. Both parameters were compared to their respective wild type model values to check for voltage shift (Vh – Vh_wt) and slope change (s – s_wt).

Inactivation: We simulated a variable voltage pre-pulse of 300 ms from -160 mV to 0 mV from a resting potential of -110 mV, followed by a 20-ms pulse to -20 mV. Then, we extracted peak currents elicited during the second pulse, normalized the data to their maximum value and plotted them against pre-pulse potential. Finally, we fitted the curve to a Boltzmann equation as in ACT and extracted Vh and slope shifts.

Mean inactivation time constants: Inactivation current time courses from the ACT protocol were isolated (from maximum peak current to the end of the pulse) and fitted to a single exponential function such as:

$$I = A * e^{-\frac{t}{\tau}} + c$$

Where I is the current, t is the time and A and c are constants. The resulting τ values were plotted against pulse potential.

Current-voltage relationship: We applied a variable voltage 250-ms pulse from -80 mV to 40 mV from a resting potential of -110 mV. Elicited peak currents were extracted, normalized to their maximum value and plotted against pulse potential.

APD_{90} Prolongation: We ran 40-beat simulations with the heterozygous SCN5A V411M isolated endocardial model (50% wild type and 50% mutated currents), saving the last beat for further analysis. Prolongation of the APD_{90} was measured relative to the wild type model. We used a target prolongation of 16% as a surrogate of the QTc prolongation.

qNaf: the total charge carried by I_{Naf} was taken into account during the APD_{90} prolongation simulations by comparing it to the wild type value. This prevented the model from not depolarizing.

1.3.3 Optimization of Flecainide

Optimization of flecainide model consisted of the following tests: Steady-state availability (SSA), recovery from use-dependent block (RUDB), I_{Naf} concentration and use-dependent block curves at 0.2, 1 and 3 Hz (Penniman et al., 2010) (I_{Naf}02, I_{Naf}1 and I_{Naf}3, respectively), I_{NaL} IC_{50} (Matsukawa et al., 2019) (I_{NaL}), and APD_{90} prolongation (Prol) (DailyMed).

Before starting the optimization, the isolated endocardial cell model was simulated for 300s at 1Hz pacing rate in drug-free conditions to reach steady state.

Flecainide enhances the inward rectifier current (I_{K1}) as found by Caballero et al. 2010 (Caballero et al., 2010), and it is estimated that 1.5 µM therapeutic flecainide (DailyMed) should increase its conductance to a 151%. Flecainide also blocks the rapid potassium delayed rectifier current (I_{Kr}) with an IC_{50} of 3.91 µM (Paul et al., 2002), which reduces the its conductance to a 72.27%.

Simulations trying to assess flecainide's I_{NaL} IC_{50} gave very high (around 90 µM) values (not shown) before optimization, revealing a lack of I_{NaL} block at therapeutic concentrations. Therefore, normal and bursting state affinities of the charged drug for the channel, but not diffusion nor neutral drug

affinities, were allowed to change during the optimization to enable fitting of the fast and late currents block dynamics.

Steady-State Availability: We used the same protocol as in the wild-type model to obtain a similar curve under the effects of 10 µM flecainide.

Recovery from use-dependent block: First, we simulated a train of 100 pulses at 25 Hz from -100mV to -10mV. Then, a second identical pulse was applied after a variable time from 0.5 to 9s at -100mV. Concentrations of flecainide were fixed at 10µM. Peak currents elicited from the delayed pulse were extracted, normalized to tonic block and plotted against time intervals.

I_{Naf} block curves: The standard protocol consisted of a 30-ms pulse from -100 mV to -20 mV. This protocol was applied 40 times at a rate of 0.2Hz or 60 times at a rate of 1 or 3 Hz. The maximum peak current elicited by the last pulse was extracted for increasing flecainide concentrations and normalized to drug-free conditions.

I_{NaL} IC_{50}: From a -120 mV 200 ms pulse, we applied a 40-ms pre-pulse to -15 mV followed by a 200-ms pulse to 40 mV. We measured I_{NaL} as the maximum elicited current during a ramp from 40 to -95 mV (-1.35 V/s) following the beforementioned pulses. The protocol was repeated 3 times for each of several drug concentrations until 50% block of the control current (drug-free) was reached. The corresponding concentration was retrieved as the I_{NaL} IC_{50}.

APD_{90} Prolongation: We performed simulations with the single cell model to test the effects of flecainide every iteration. We applied a train of 40 beats at 1 Hz with 1.5 µM therapeutic flecainide. APD_{90} from the last beat was normalized to drug free conditions to obtain drug-induced prolongation. Target prolongation was adjusted to be as low as possible.

Because flecainide has a pKa of 9.3 (Moreno et al., 2011) it is 99% charged under physiological conditions, before optimizing the complete drug model – in a first phase – we optimized only neutral flecainide for DDUDB (see below) at 10 Hz and RUDB for time intervals ranging from 0.5s to 7s. Then, neutral drug parameters were held constant while the remaining parameters were optimized using all protocols in two additional phases. The second phase included the complete drug versions of the abovementioned protocols as well as SSA, RUDB and the I_{Naf}02, I_{Naf}1 and I_{Naf}3 set. Finally, the third phase added I_{NaL} and APD_{90} prolongation in wild-type to the test batch.

Dose-dependent use-dependent block for neutral flecainide (DDUDB): We simulated a train of 300, 25-millisecond, pulses at 10Hz from -100mV to -10mV. We normalized the current elicited by the last pulse under increasing neutral flecainide concentrations and normalized the value to the first pulse. We plotted the results against drug concentration.

Flecainide optimizations were terminated as usual when the conditions were met. Nonetheless, to improve the I_{NaL} IC_{50}, we searched for best value in the parameter history giving similar fitting results in the other tests.

1.3.4 Optimization of ranolazine

Optimization of ranolazine consisted of the following test protocols: steady-state availability (SSA), tonic block of peak and late sodium current (TB), use-dependent block (UDB), recovery from use-dependent block (RUDB) and frequency-dependent use-dependent block (FDUDB). Diffusion and

affinities for the normal and bursting states were set as in the original model and prevented from being modified.

Steady-state availability: we applied a 100 ms pulse to -10 mV from a variable conditioning potential of -120 mV to -40 mV and extracted the resulting peak current under exposure to 10 µM ranolazine. Values were normalized to drug-free conditions and plotted against conditioning pulse potential.

Tonic block: A single 500-millisecond pulse to -10 mV from a resting potential of -100 mV was applied to evaluate the effect of increasing ranolazine concentrations. We extracted I_{Naf} as the elicited peak current, while I_{NaL} was measured as the remaining current at the end of the pulse. Both values were normalized to drug-free conditions and plotted against drug concentrations.

Use-dependent block: We applied 300, 25 ms, pulses to -10 mV from a resting potential of -100 mV at a rate of 5 Hz and under exposure to increasing ranolazine concentrations. We saved the peak current elicited by the last pulse, normalized it to drug-free conditions and plotted the resulting values against drug concentrations.

Recovery from use-dependent block: This protocol is similar to flecainide’s RUDB protocol. Intervals ranged from 0.1 s to 10 s and ranolazine concentrations were set to 10 µM.

Frequency-dependent use-dependent block: A train of 300 square 25-ms pulses from -100 mV to -10 mV were applied at 1 Hz, 2 Hz, 5 Hz and 10 Hz rates under exposure to 100 µM ranolazine. The peak sodium currents elicited by the last pulse were extracted and normalized to drug-free conditions, then plotted against pacing rate.

2 Supplementary Figures

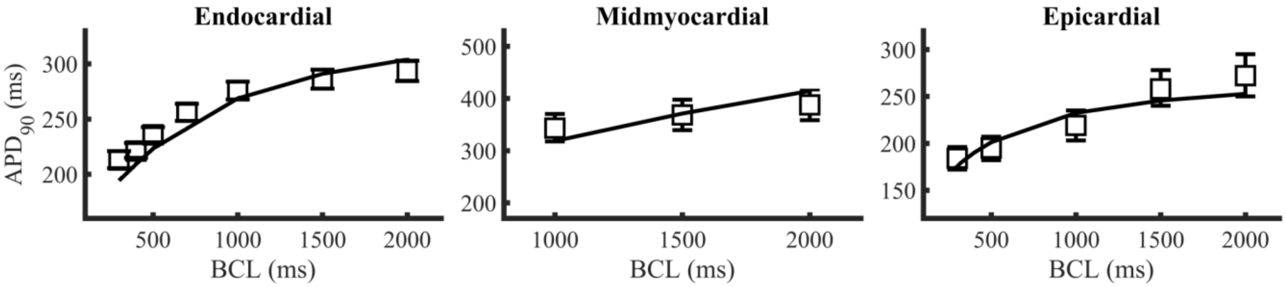

Supplementary Figure S1. Simulated wild-type isolated endocardial (left), midmyocardial (middle) and epicardial (right) steady-state APD_{90} restitution curves. Lines are simulations and squares are reference data (mean ± SD bars) from O'Hara and coworkers (O'Hara et al., 2011).

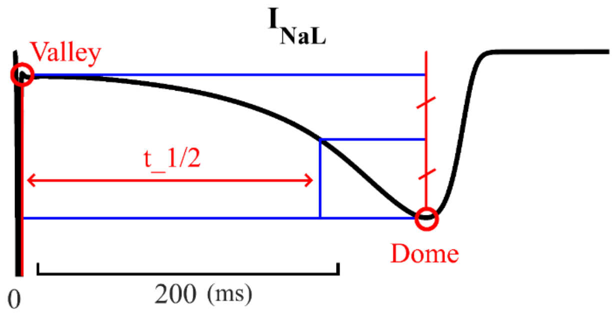

Supplementary Figure S2. Calculation of the three parameters we used to evaluate the time course of I_{NaL} (dome, valley and t_1/2). Valley was normalized to the dome value, while t_1/2 was normalized to the total time between dome and valley.

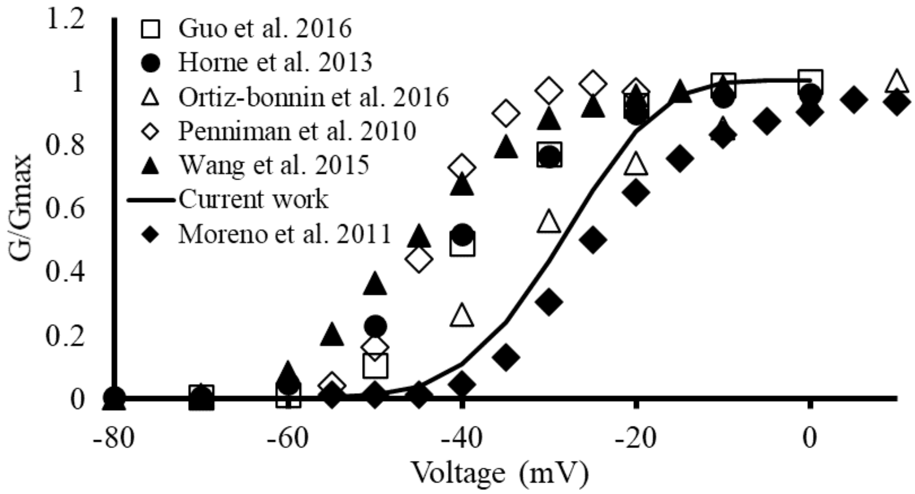

Supplementary figure S3. Comparison between SCN5A sodium channel activation dynamics from different sources (Penniman et al., 2010; Horne et al., 2011; Moreno et al., 2011; Wang et al., 2015; Guo et al., 2016; Ortiz-Bonnin et al., 2016) (symbols) and our wild type model (black line). The reference we used to fit the model was Moreno et al. 2011.

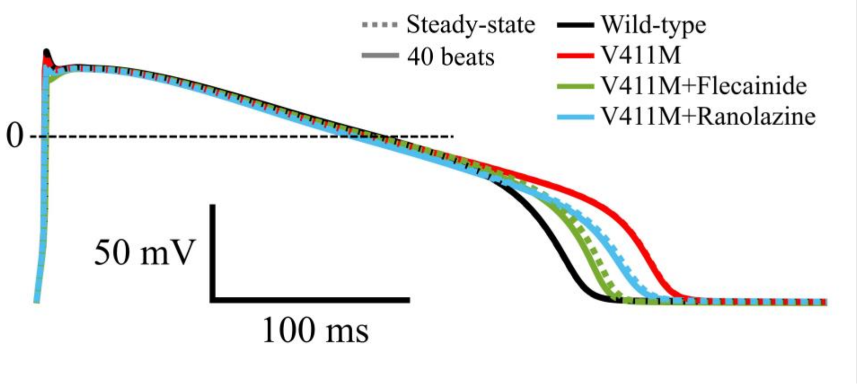

Supplementary Figure S4. Comparison of the simulated action potential time courses for isolated endocardial cells after a train of 40 pulses (continuous lines) and at the steady-state (300 pulses, dashed lines). Wild type (black lines) and V411M mutated (red lines) cells in control and under exposure to therapeutic concentrations of flecainide (green lines) and ranolazine (blue lines). The very small differences between the simulated action potential time courses obtained with 40 and 300 pulses corroborate the validity of applying 40 stimuli to the isolated endocardial models in order to reduce the computational cost during the optimization procedure of the wild type, flecainide and mutation I_{Na} models.

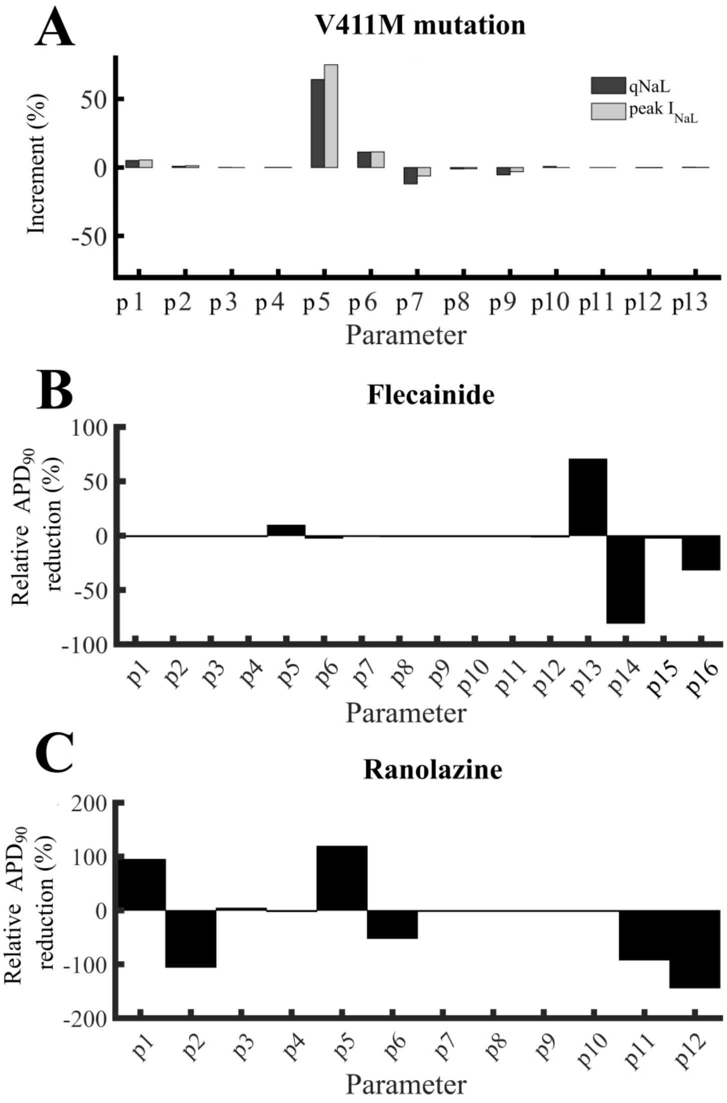

Supplementary Figure S5. Sensitivity analysis of V411M I_{Na} (A), flecainide (B) and ranolazine (C) model parameters using the isolated endocardial cellular model. A: Peak I_{NaL} and qNaL increments normalized to the values obtained with the optimized model of the V411M mutation when only considering one parameter of the V411M model at a time. Parameters p1 to p13 are defined in Supplementary Table T2. B: APD_{90} reduction relative to the one produced by the flecainide model in the presence of the V411M mutation when multiplying by 10 one of the optimized flecainide parameters at a time. For example, multiplying p13 by 10 further reduced the APD_{90} of the mutated cell by an additional 70.5% compared to the reduction exerted by flecainide. Parameters p1 to p16 are defined in supplementary table T3. C: Ranolazine sensitivity analysis was performed in a similar way as for flecainide. For example, multiplying p2 by 10 prevented ranolazine from shortening the APD_{90}. Parameters p1 to p12 are defined in supplementary table T4.

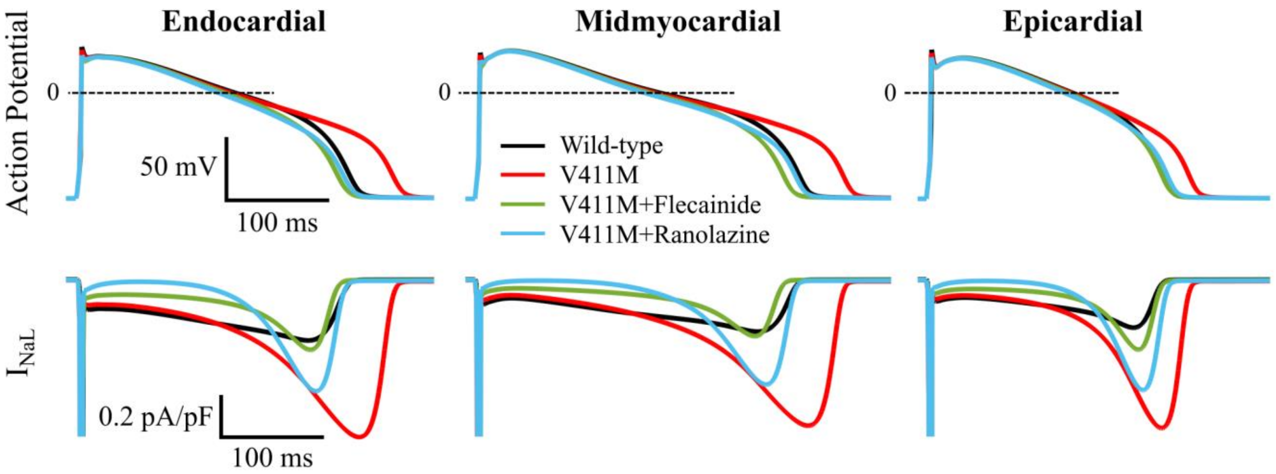

Supplementary Figure S6. Simulated action potential (top row) and I_{NaL} (bottom row) steady-state time courses of the isolated endocardial (left column), midmyocardial (middle column) and epicardial (right column) cells under exposure to therapeutic concentrations of flecainide (green) and ranolazine (blue) without accounting for the effects on I_{Kr} and I_{K1}. The APD_{90}s of the action potential time courses under flecainide and ranolazine treatments in those conditions were, respectively, 257.5 ms and 263.3 ms for the endocardial cells, 303.7 ms and 315.5 ms for the midmyocardial cells, and 232 ms and 237.3 ms for the epicardial cells. The heterozygous V411M mutant (red) and wild type (black) time courses were also added for comparison.

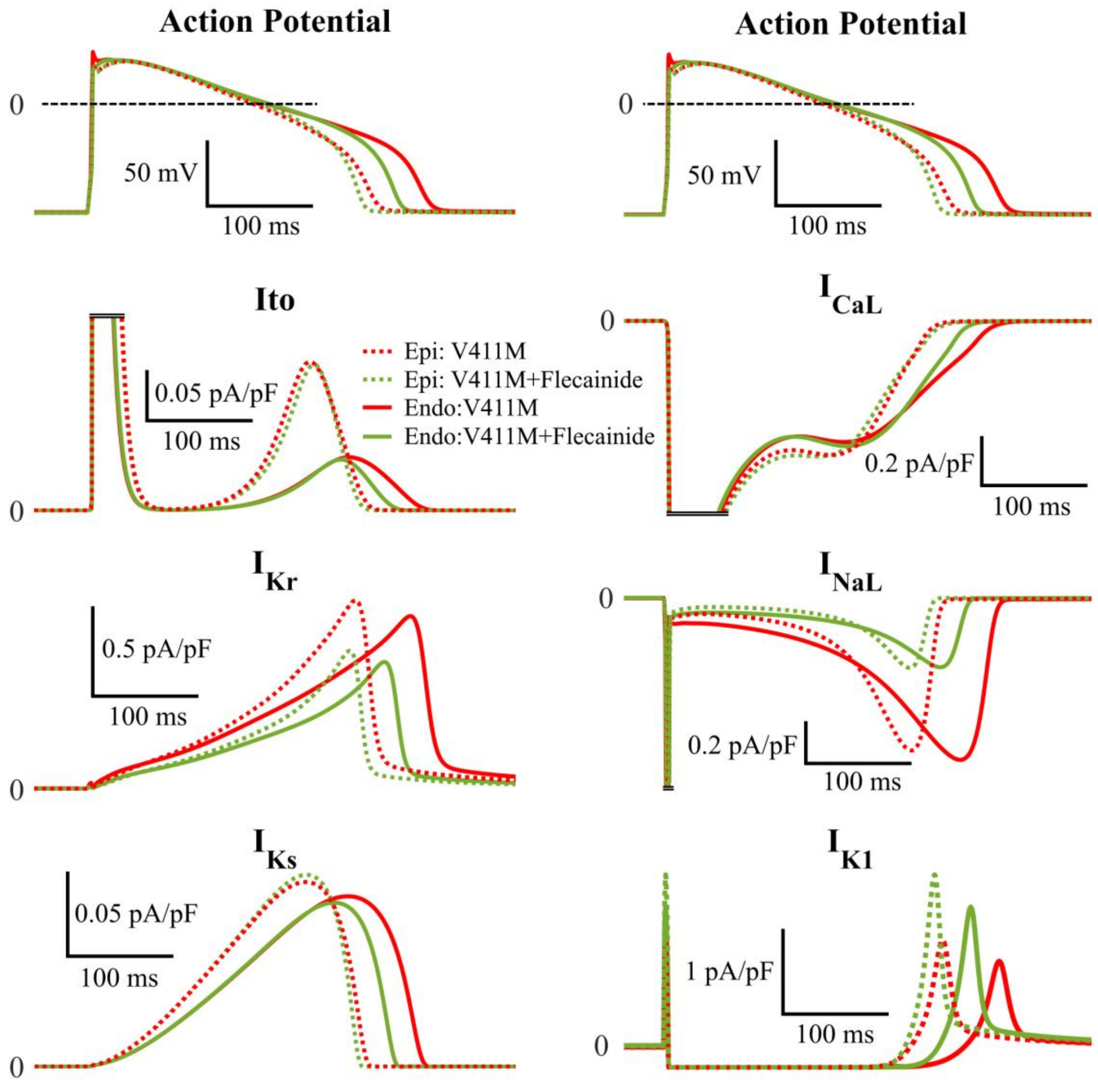

Supplementary Figure S7. Comparison of the time courses of the action potential and main currents of the isolated epicardial (dashed lines) and endocardial (continuous lines) mutant cells in drug-free (red lines) and under exposure to therapeutic concentrations of flecainide (green lines). Action potential time courses (top panels) were included in both columns for reference. Repolarizing current time courses include the transient outward potassium current (Ito), the rapid and slow components of the delayed rectifier potassium currents (I_{Kr} and I_{Ks}) and the potassium rectifier current (I_{K1}). Depolarizing current time courses include the type-L calcium current (I_{CaL}) and the late sodium current (I_{NaL}).

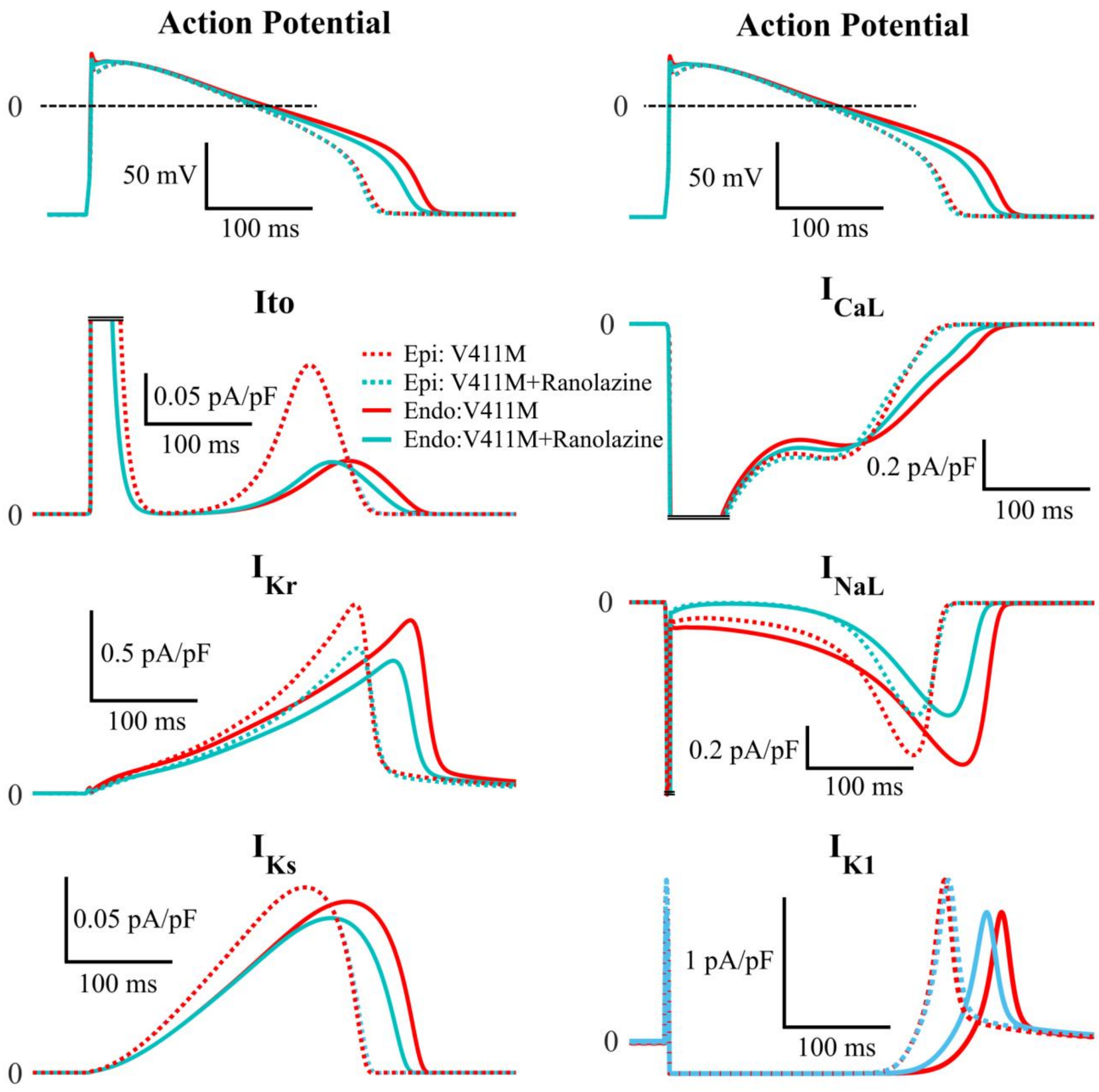

Supplementary Figure S8. Comparison of the time courses of the action potential and main currents of the isolated epicardial (dashed lines) and endocardial (continuous lines) mutant cells in drug-free (red lines) and under exposure to therapeutic concentrations of ranolazine (blue lines). Action potential time courses (top panels) were included in both columns for reference. Repolarizing current time courses include the transient outward potassium current (Ito), the rapid and slow components of the delayed rectifier potassium currents (I_{Kr} and I_{Ks}) and the potassium rectifier current (I_{K1}). Depolarizing current time courses include the type-L calcium current (I_{CaL}) and the late sodium current (I_{NaL}).

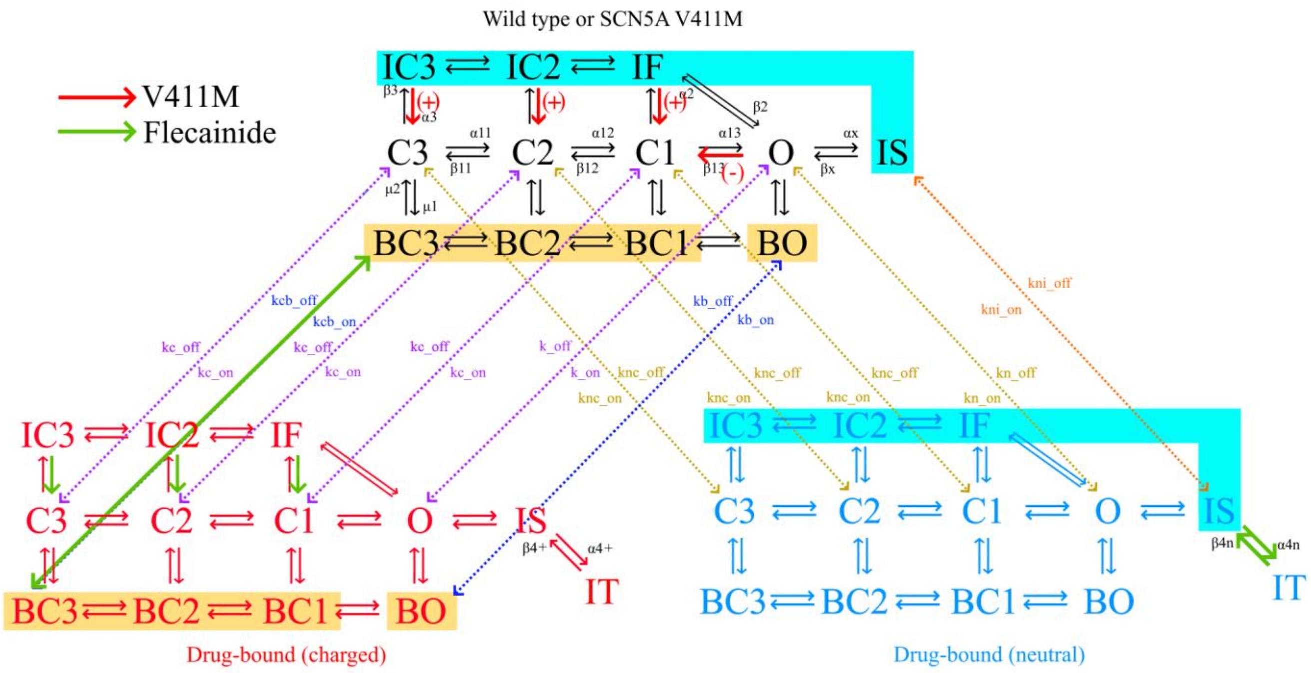

Supplementary Figure S9. Most important rates defining the effects of flecainide (thick green arrows) on the sodium channel Markov model. The changes that were induced by the V411M mutation were also indicated as thick red arrows. A plus sign indicates an increase of the rate, while a minus sign indicates the opposite. See Figure 2 caption for more details about the diagram of the sodium channel Markovian model.

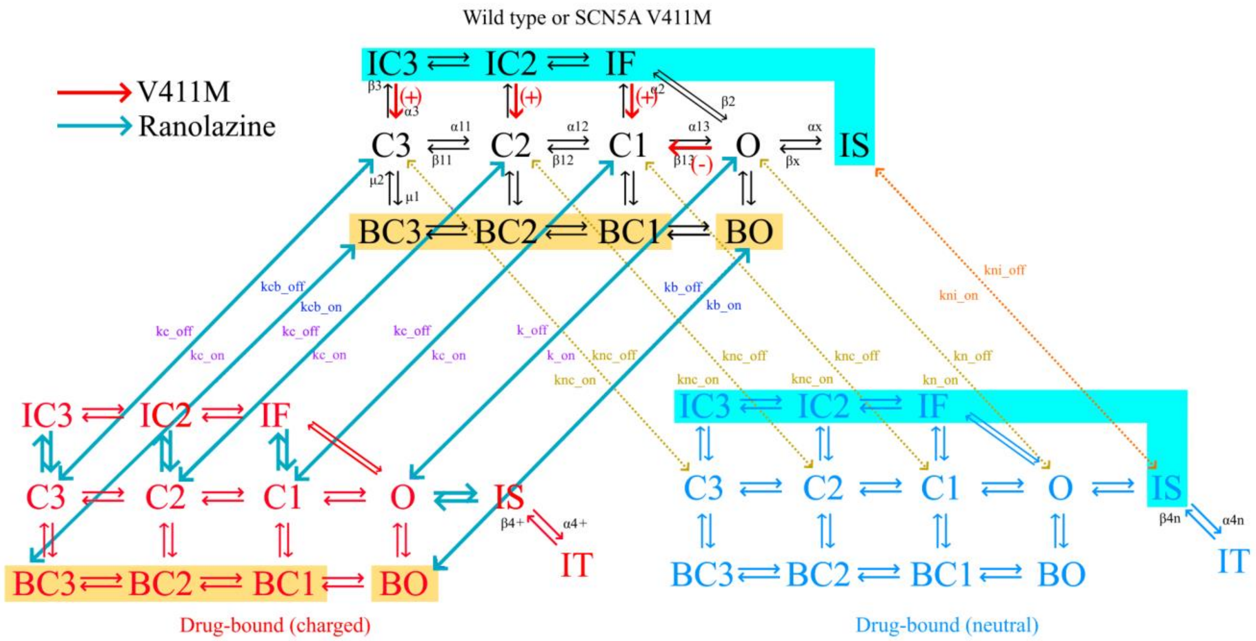

Supplementary Figure S10. Most important rates defining the effects of ranolazine (thick blue arrows) on the sodium channel Markov model. The changes that were induced by the V411M mutation were also indicated as thick red arrows. A plus sign indicates an increase of the rate, while a minus sign indicates the opposite. See Figure 2 caption for more details about the diagram of the sodium channel Markovian model.

3 Supplementary tables

Supplementary Table T1. Conductance relationship between several currents of the isolated endocardial, midmyocardial and epicardial action potential models. The first column indicates the abbreviation of the current conductance name, and the second and third columns represent the scaling factors applied to the endocardial conductances to generate midmyocardial and epicardial action potential cellular models, respectively. As the I_{NaL} conductance (G_{NaL}) does not exist in our model, the corresponding factor was applied to µ1, a transition velocity that governs the transition from normal to bursting states (see Figure 2 of the main text).

Conductance	Midmyocardial	Epicardial
G_{Kr}	0.75	1.1
G_{Ks}	1.4	1
G_{CaL}	1.80	1.10
G_{NaK}	0.70	0.90
G_{to}	3	3
G_{K1}	1.3	1.2
G_{NCX}	1.4	1.1
G_{NaL}	0.9	0.6

Supplementary Table T2. Equations of the transition rates from the wild type and V411M I_{Na} models. P1 to p13 are the parameters fitted during the optimizations and they were restricted to positive values. Tf is the temperature factor and v is the membrane voltage.

Transition rates (ms^{-1})

$$\alpha 11 = Tf \cdot \frac{8.5539}{(\mathbf{p1}) \cdot e^{-\frac{v}{17.0}} + (\mathbf{p2}) \cdot e^{-\frac{v}{150}}}$$

$$\alpha 12 = Tf \cdot \frac{8.5539}{(\mathbf{p1}) \cdot e^{-\frac{v}{15.0}} + (\mathbf{p2}) \cdot e^{-\frac{v}{150}}}$$

$$\alpha 13 = Tf \cdot \frac{8.5539}{(\mathbf{p1}) \cdot e^{-\frac{v}{12.0}} + (\mathbf{p2}) \cdot e^{-\frac{v}{150}}}$$

$$\beta 11 = Tf \cdot (\mathbf{p3}) \cdot e^{-\frac{v}{20.3}}$$

$$\beta 12 = Tf \cdot (\mathbf{p4}) \cdot e^{-\frac{v-5}{20.3}}$$

$$\beta 13 = Tf \cdot (\mathbf{p5}) \cdot e^{-\frac{v-10}{20.3}}$$

$$\alpha 3 = Tf \cdot (\mathbf{p6}) \cdot e^{-\frac{v}{(\mathbf{p7})}}$$

$$\beta 3 = Tf \cdot (\mathbf{p8}) \cdot e^{\frac{v}{(\mathbf{p9})}}$$

$$\alpha 2 = Tf \cdot (\mathbf{p10}) \cdot e^{\frac{v}{(\mathbf{p11})}}$$

$$\beta 2 = \frac{\alpha 13 \cdot \alpha 2 \cdot \alpha 3}{\beta 13 \cdot \beta 3}$$

$$\beta x = (\mathbf{p12}) \cdot \alpha 3$$

$$\alpha x = (\mathbf{p13}) \cdot \alpha 2$$

$$\mu 1 = 1.70 \cdot 10^{-7}$$

$$\mu 2 = 5.66 \cdot 10^{-4}$$

Supplementary Table T3. Transition rates and affinities of the flecainide model. P1 to p16 are the parameters that were fitted during the optimizations and they were restricted to positive values.

Transition rates (ms^{-1})	equations
α11+ and α11n	$\alpha 11$
α12+ and α12n	$\alpha 12$
β11+ and β11n	$\beta 11$
β12+ and β12n	$\beta 12$
αx+	$(\boldsymbol{p1}) \cdot \alpha x$
βx+	$(\boldsymbol{p2}) \cdot \beta x$
α13+	$(\boldsymbol{p3}) \cdot \alpha 13$
α2+	$(\boldsymbol{p4}) \cdot \alpha 2$
β3+	$(\boldsymbol{p5}) \cdot \beta 3$
α3+	$(\boldsymbol{p6}) \cdot \alpha 3$
α4+	$(\boldsymbol{p7}) \cdot \alpha 2$
β4+	$(\boldsymbol{p8}) \cdot \alpha 3$
αxn	$(\boldsymbol{p9}) \cdot \alpha x$
α13n	$(\boldsymbol{p10}) \cdot \alpha 13$
α2n	$(\boldsymbol{p11}) \cdot \alpha 2$
β3n	$(\boldsymbol{p12}) \cdot \beta 3$
α4n	$(\boldsymbol{p13}) \cdot \alpha 2$
β4n	$(\boldsymbol{p14}) \cdot \alpha 3$
k_on = kc_on	$drug_{charged} \cdot diffusion$
k_off = kc_off	$(\boldsymbol{p15}) \cdot 10^{-6} \cdot e^{\frac{-0.7*V*F}{R*T}} * diffusion$
kb_on = kcb_on	k_on
kb_off = kcb_off	$(\boldsymbol{p16}) \cdot 10^{-6} \cdot e^{\frac{-0.7*V*F}{R*T}} * diffusion$
kn_on	$drug\_neutral \cdot diffusion$
kn_off	$400 \cdot 10^{-6} \cdot diffusion$
kni_on	kn_on
kni_off	$5.4 \cdot 10^{-6} \cdot diffusion$
knc_on	kn_on
knc_off	$800 * 10^{-6} \cdot diffusion$
diffusion	$5500\ M^{-1} \cdot ms^{-1}$

Supplementary Table T4. Transition rates and affinities of the ranolazine model. P1 to p10 are the parameters fitted during the optimizations and they were restricted to positive values. P11 and p12 were incorporated only for explaining the supplementary Figure S5, but they were not modified during the optimization of the ranolazine model.

Transition rates (ms^{-1})	equations
α11+ and α11n	$\alpha 11$
α12+ and α12n	$\alpha 12$
β11+ and β11n	$\beta 11$
β12+ and β12n	$\beta 12$
αx+	$(\boldsymbol{p1}) \cdot \alpha x$
βx+	$(\boldsymbol{p2}) \cdot \beta x$
α13+	$(\boldsymbol{p3}) \cdot \alpha 13$
α2+	$(\boldsymbol{p4}) \cdot \alpha 2$
β3+	$(\boldsymbol{p5}) \cdot \beta 3$
α3+	$(\boldsymbol{p6}) \cdot \alpha 3$
αxn	$(\boldsymbol{p7}) \cdot \alpha x$
α13n	$(\boldsymbol{p8}) \cdot \alpha 13$
α2n	$(\boldsymbol{p9}) \cdot \alpha 2$
β3n	$(\boldsymbol{p10}) \cdot \beta 3$
k_on = kc_on	$drug_{charged} \cdot diffusion$
k_off = kc_off	$(\boldsymbol{p11}) \cdot 10^{-6} \cdot e^{\frac{-0.7*V*F}{R*T}} * diffusion$
kb_on = kcb_on	k_on
kb_off = kcb_off	$(\boldsymbol{p12}) \cdot 10^{-6} \cdot e^{\frac{-0.7*V*F}{R*T}} * diffusion$
kn_on	$drug\_neutral \cdot diffusion$
kn_off	$400 \cdot 10^{-6} \cdot diffusion$
kni_on	kn_on
kni_off	$5.4 \cdot 10^{-6} \cdot diffusion$
knc_on	kn_on
knc_off	$800 * 10^{-6} \cdot diffusion$
diffusion	$5500\ M^{-1} \cdot ms^{-1}$

Supplementary Table T5. I_{Na} wild-type model optimization error calculations and weights in the cost function. The words containing “data” and “reference” indicate vectors containing the results of our tests (black lines in Figure 3 of the main text) and the reference experimental values (open squares in Figure 3 of the main text), respectively. N is the number of samples. APD refers to a vector containing the APD_{90}s of simulations at BCLs 300, 400, 500, 1000, 1500 and 2000 ms. Mean open time was adjusted to 0.5 at V=-30 mV.

Wild type optimization

**Test name**	**Error calculation**	**Weight**
Steady state availability	(100·sum(data-reference))^2/N	2
Activation	(100·sum(data-reference))^2/N	2
Recovery from inactivation	(100·sum(data-reference))^2/N	1
Recovery from use-dependent block	(100·sum(data-reference))^2/N	0.04
Mean opening time	abs(2 - α2+β13+αx)	1
Tau 50% activation	(100·sum(data-reference))^2/N	1
INaL time course at BCL 1000 ms	100·((dome-reference_dome)^2+(valley-reference_valley)^2+(time_50-reference_time50))	5
APD90 tests (at BCL 300, 400, 500, 1000, 1500 and 2000 ms)	sum(APD_data-APD_reference)^2	BCL 500 ms: 2.5<br>Others: 1
Max dVdt	sum(dVdt_data-dVdt_reference)^2	1
INaL current-voltage relationship	(100·sum(data-reference))^2/N	1

Supplementary Table T6. I_{Na} V411M mutation model optimization error calculations and weights in the cost function. The words containing “data” and “reference” indicate vectors containing the results of our tests (red lines in Figure 4 of the main text) and the reference target values (open squares in Figure 4 of the main text), respectively. N is the number of samples.

V411M mutation optimization

**Test name**	**Error calculation**	**Weight**
Activation	((Vh_data-Vh_reference)^2+ (slope_data-slope_reference)^2)/2	1
Activation time constant (tau)	sum(Tau_data-tau_reference)^2/N	Positions 4 to 8: 5 Others: 1
Inactivation	((Vh_data-Vh_reference)^2+(slope_data-slope_reference)^2)/2	10
current-voltage relationship	(100·sum(data-reference))^2/N	Positions 6 to 9 from both ends: 2 RestOthers: 1
Prolongation in endocardial cell (%)	(Prolongation_data-Prolongation_reference)^2	2.5
qNaf (to prevent no depolarization)	10^6·(WT_qNaf-V411M_qNaf)^2	2

Supplementary Table T7. Flecainide model optimization error calculations and weights in the cost function. The words containing “data” and “reference” indicate vectors containing the results of our tests (dots and black lines in Figure 5 of the main text) and the reference experimental values (open squares in Figure 5 of the main text), respectively. The INaL IC50 test was not shown. N is the number of samples.

Flecainide optimization

**Test name**	**Error calculation**	**Weight**
Steady state availability	(100·sum(data-reference))^2/N	2
Dose-dependent use-dependent block	(100·sum(data-reference))^2/N	1
Recovery from use-dependent block	(100·sum(data-reference))^2/N	1
Frequency-dependent INaf block at 0.2 Hz	(100·sum(IC50_data-IC50_reference))^2/N	1
Frequency-dependent INaf block at 1 Hz	(100·sum(IC50_data-IC50_reference))^2/N	1
Frequency-dependent INaf block at 3 Hz	(100·sum(IC50_data-IC50_reference))^2/N	1
Prolongation in endocardial cell (%)	10·(Prolongation)^2	1
INaL IC50	1000·(INaL_IC50-INaL_IC50_reference)^2	1

Supplementary Table T8. Ranolazine model optimization error calculations and weights in the cost function. The words containing “data” and “reference” indicate vectors containing the results of our tests (black lines in Figure 6 of the main text) and the reference experimental values (open squares in Figure 6 of the main text), respectively. N is the number of samples.

Ranolazine optimization

**Test name**	**Error calculation**	**Weight**
Steady state availability	(100·sum(data-reference))^2/N	1
Tonic block of INaf	(100·sum(data-reference))^2/N	1
Tonic block of INaL	(100·sum(data-reference))^2/N	1
Recovery from use-dependent block	(100·sum(data-reference))^2/N	1
Frequency-dependent use-dependent block	(100·sum(data-reference))^2/N	1

4 References

Caballero, R., Dolz-Gaiton, P., Gomez, R., Amoros, I., Barana, A., Gonzalez de la Fuente, M., et al. (2010). Flecainide increases Kir2.1 currents by interacting with cysteine 311, decreasing the polyamine-induced rectification. *Proc. Natl. Acad. Sci.* 107, 15631–15636. doi:10.1073/pnas.1004021107.

DailyMed Available at: https://dailymed.nlm.nih.gov/dailymed/index.cfm.

Dutta, S., Chang, K. C., Beattie, K. A., Sheng, J., Tran, P. N., Wu, W. W., et al. (2017). Optimization of an In silico Cardiac Cell Model for Proarrhythmia Risk Assessment. *Front. Physiol.* 8, 616. doi:10.3389/fphys.2017.00616.

Guo, Q., Ren, L., Chen, X., Hou, C., Chu, J., Pu, J., et al. (2016). A novel mutation in the SCN5A gene contributes to arrhythmogenic characteristics of early repolarization syndrome. *Int. J. Mol. Med.* 37, 727–33. doi:10.3892/ijmm.2016.2468.

Hegyi, B., Bányász, T., Izu, L. T., Belardinelli, L., Bers, D. M., and Chen-Izu, Y. (2018). β-adrenergic regulation of late Na+ current during cardiac action potential is mediated by both PKA and CaMKII. *J. Mol. Cell. Cardiol.* 123, 168–179. doi:10.1016/j.yjmcc.2018.09.006.

Horne, A. J., Eldstrom, J., Sanatani, S., and Fedida, D. (2011). A novel mechanism for LQT3 with 2:1 block: a pore-lining mutation in Nav1.5 significantly affects voltage-dependence of activation. *Hear. Rhythm* 8, 770–7. doi:10.1016/j.hrthm.2010.12.041.

Horvath, B., Banyasz, T., Jian, Z., Hegyi, B., Kistamas, K., Nanasi, P. P., et al. (2013). Dynamics of the late Na(+) current during cardiac action potential and its contribution to afterdepolarizations. *J. Mol. Cell. Cardiol.* 64, 59–68. doi:10.1016/j.yjmcc.2013.08.010.

Matsukawa, H., Izumi, T., Yamaguchi, M., Tomizawa, S., and Ohshiro, H. (2019). Safety Pharmacology Assessment of Cardiac Ion Channels by Manual Patch Clamp With CiPA Protocols. Poster session presented at 2019 annual meeting of Safety Pharamacology Society, Barcelona, ES. September.

Moreno, J. D., Lewis, T. J., and Clancy, C. E. (2016). Parameterization for In-Silico Modeling of Ion Channel Interactions with Drugs. *PLoS One* 11, e0150761. doi:10.1371/journal.pone.0150761.

Moreno, J. D., Yang, P.-C., Bankston, J. R., Grandi, E., Bers, D. M., Kass, R. S., et al. (2013). Ranolazine for congenital and acquired late INa-linked arrhythmias: in silico pharmacological screening. *Circ. Res.* 113, e50–e61. doi:10.1161/CIRCRESAHA.113.301971.

Moreno, J. D., Zhu, Z. I., Yang, P.-C., Bankston, J. R., Jeng, M.-T., Kang, C., et al. (2011). A computational model to predict the effects of class I anti-arrhythmic drugs on ventricular rhythms. *Sci. Transl. Med.* 3, 98ra83. doi:10.1126/scitranslmed.3002588.

O’Hara, T., Virág, L., Varró, A., and Rudy, Y. (2011). Simulation of the undiseased human cardiac ventricular action potential: model formulation and experimental validation. *PLoS Comput. Biol.* 7, e1002061. doi:10.1371/journal.pcbi.1002061.

Ortiz-Bonnin, B., Rinné, S., Moss, R., Streit, A. K., Scharf, M., Richter, K., et al. (2016). Electrophysiological characterization of a large set of novel variants in the SCN5A-gene: identification of novel LQTS3 and BrS mutations. *Pflugers Arch. Eur. J. Physiol.* 468, 1375–1387. doi:10.1007/s00424-016-1844-3.

Paul, A. A., Witchel, H. J., and Hancox, J. C. (2002). Inhibition of the current of heterologously expressed HERG potassium channels by flecainide and comparison with quinidine, propafenone and lignocaine. *Br. J. Pharmacol.* 136, 717–29. doi:10.1038/sj.bjp.0704784.

Penniman, J. R., Kim, D. C., Salata, J. J., and Imredy, J. P. (2010). Assessing use-dependent inhibition of the cardiac Na± current (INa) in the PatchXpress automated patch clamp. *J. Pharmacol. Toxicol. Methods* 62, 107–118. doi:10.1016/j.vascn.2010.06.007.

Wang, Y., Mi, J., Lu, K., Lu, Y., and Wang, K. (2015). Comparison of Gating Properties and Use-Dependent Block of Nav1.5 and Nav1.7 Channels by Anti-Arrhythmics Mexiletine and Lidocaine. *PLoS One* 10, e0128653. doi:10.1371/journal.pone.0128653.
